# Health Effects of Metabolic Risks in the United States From 1990 to 2019

**DOI:** 10.3389/fpubh.2022.751126

**Published:** 2022-01-31

**Authors:** Ruifang Chen, Saeid Safiri, Masoud Behzadifar, Jude Dzevela Kong, Mohamed Sami Zguira, Nicola Luigi Bragazzi, Wen Zhong, Weiru Zhang

**Affiliations:** ^1^Medical Experimental Center, The Third Xiangya Hospital, Central South University, Changsha, China; ^2^Neurosciences Research Center, Aging Research Institute, Tabriz University of Medical Sciences, Tabriz, Iran; ^3^Social Determinants of Health Research Center, Lorestan University of Medical Sciences, Khorramabad, Iran; ^4^Department of Mathematics and Statistics, Centre for Disease Modelling, York University, Toronto, ON, Canada; ^5^Department of Physiology and Lung Function Testing, Faculty of Medicine Ibn-El-Jazzar, University of Sousse, Sousse, Tunisia; ^6^Department of General Medicine, Xiangya Hospital, Central South University, Changsha, China; ^7^National Clinical Research Center for Geriatric Disorders, Xiangya Hospital, Central South University, Changsha, China

**Keywords:** metabolic risk, disability adjusted life year, death, United States, global burden of disease

## Abstract

**Introduction:**

Metabolic risks including high body mass index, high fasting plasma glucose, high low-density lipoprotein cholesterol, high systolic blood pressure, kidney dysfunction and low bone mineral density, contribute heavy burden to the US health systems. We aimed to investigate the burden attributable to metabolic risks in the US from 1990 to 2019.

**Methods:**

Using methodology of Global Burden of Disease Study, the deaths and DALYs attributable to metabolic risks were analyzed by age, gender, states, Socio-demographic Index (SDI) and diseases from 1990 to 2019 in the US.

**Results:**

In 2019, the age-standardized death and DALY rates attributable to metabolic risks were 174.9 and 4738.7 per 100,000 people, accounting for 33.1% and 18.2% of death and DALY rates from all causes in the US, and there was a decrease by −32.5% and −21.2% in age-standardized death and DALY rates since 1990. The burden attributable to metabolic risks increased with age, and was higher in males than females. In addition, the burden varied widely across the states, generally in inverse proportion to the SDI levels, and the heaviest burden was observed in East and West South-Central of the US. Cardiovascular diseases carried heavy burden attributable to metabolic risks.

**Conclusion:**

The burden attributable to metabolic risks remained major public health concerns in the US. Prevention of metabolic risks should be a high priority in the US.

## Introduction

Metabolic risks, including high body mass index (BMI), high fasting plasma glucose (FPG), high low-density lipoprotein (LDL) cholesterol, high systolic blood pressure (SBP), kidney dysfunction and low bone mineral density (BMD), are important modifiable risk factors for a wide range of diseases, such as cardiovascular diseases, cancers and injuries (1). In the US, most of the 10 leading causes in 2019 was attributable to these metabolic risks (2), which posed threaten to public health, and it was reported that in 2017 the age-standardized death and DALYs rate of high fasting plasma glucose were 70.97 and 1855.74 per 100,000 (3), and 72.0 and 2355.1 per 100,000 for high BMI in the US (4). However, the US healthcare system has focused mainly on drug discoveries and disease treatment rather than prevention (5). According to data from NHANES, the age-adjusted prevalence of obesity (BMI ≥ 30 kg/m^2^), high blood pressure (SBP ≥ 130 mmHg or DBP ≥ 90 mmHg), and high total cholesterol (≥240 mg/dL) in adults aged over 20 was 42.4, 47.1, 14.4, and 10.5% in 2017, respectively (6), and 6.7% for chronic kidney dysfunction in 2016 (7), 43.9% in adults aged over 50 for low BMD in 2010 (8), which reflected high exposures to metabolic risks in the US residents, bringing out huge burden to health system, for instance, high BMI brought about 216,000 deaths, and costed about 3.38–6.38 billion US dollar annually (1, 4, 9, 10).

Along with the unbalanced change of social development, epidemiology and demographic within the US, the representative nation of high incomes countries in North America, risk factors still have had divergent trajectories in each state (9). Considering crucial impacts on occurrence and development of diseases, cartography of the disparities will facilitate the management of metabolic risks. Although there were some studies having discussed the prevalence or burden of BMI, diabetes or high cholesterol in the US (9, 11), long-term systemic, consistent and comparable results were still scarce. Based on the up-to-date iteration of Global Burden of Disease (GBD) study 2019, our work will provide a detailed information on the mortality and morbidity attributable to metabolic risks by age, gender, state, Socio-demographic Index (SDI) and causes from 1990 to 2019 in the US.

## Method

### Data Source

The GBD study was conducted by the Institute of Health Metrics and Evaluation to provide annual reports on the burden of diseases, injuries, and risk factors. GBD 2019 focused on 369 diseases, injuries, and 87 risk factors systematically. The general methods used in GBD 2019 have been published previously (1, 12, 13). Our study included the residents of all age groups in the US from 1990 to 2019. Data sources used to estimate the DALYs and deaths attributable to individual and combined effects of metabolic risks were extracted by GBD 2019 Data Input Sources Tool (http://ghdx.healthdata.org/gbd-2019/data-input-sources), and the results were available through online query tools on the website (http://ghdx.healthdata.org/gbd-results-tool). Since no identifiable data were used, and the patients and public were not involved in collecting the data or determining the research question, outcome measures, and study design, ethics approval and informed consent were not required for this study.

### Definition of Metabolic Risks

TMREL (the theoretical minimum-risk exposure level) of a certain risk factor was defined as the minimum level for death or DALY -weighted multi-cause curves of the risk factor from previous meta-analysis studies. PAF is a term used in GBD, that is the proportion by which the outcome would be reduced in a given population and in a given year if the exposure to a risk factor in the past were reduced to the counterfactual level of the TMREL (1). TMREL of high BMI was defined as BMI ≥ 25 kg/m^2^ for adults (ages 20+), and was based on International Obesity Task Force standards for children (ages 1–19) (14). High FPG was defined as any level above the TMREL (4.8–5.4 mmol/L). Although FPG ≥ 7.0 mmol/L is the accepted definition for diabetes, this threshold cannot capture all the increased risk caused by disturbance of FPG (15). Therefore, based on prospective cohort studies, the TMREL of FPG was estimated to 4.8–5.4 mmol/L, avoiding exposing a large proportion of the population to increased risk of mortality (16). Similarly, high LDL cholesterol was defined as LDL-cholesterol above TMREL level (0.7–1.3 mmol/L) (17). High SBP was defined as SBP above TMREL level (110–115 mmHg), because although the majority of the burden associated with SBP occurred in persons with hypertension (SBP ≥ 140 mm Hg), there was still 30% occurred in individuals with an SBP between 110 and 140 mm Hg (18). Kidney dysfunction was defined by urinary albumin to creatinine ratio and estimated glomerular filtration rate as KDIGO classification (CKD stage 1–5) (19). Since bone mineral varied with age and gender, the TMREL of low BMD was defined as the age-sex specific 99th percentile of BMD based on NHANES study (1).

### Estimation of Burden

The burden attributable to metabolic risks was measured by deaths and DALYs. Deaths attributable to metabolic risks were calculated by the Cause of Death Ensemble Model, and predictive covariates were used to develop a series of the best plausible models to estimate of deaths due to metabolic risks by location, age, sex, and year. DALYs represented the sum of years of life lost (YLLs) and years lived with disability (YLDs). YLLs were calculated by multiplying of deaths attributed to metabolic risks by the life expectancy, and YLDs were the health loss associated with severity levels, ranging from 0 (complete health) to 1 (death) (12). DALYs attributable to metabolic risks were estimated using DisMod-MR, a Bayesian meta-regression disease model for non-fatal health outcomes in GBD study (12, 13). Then, deaths and DALYs were quantified using the GBD comparative risk assessment framework. For each risk exposure–disease outcome pairs, they were firstly ascertained with sufficient evidences from prospective cohorts or case-control studies, and PAF of the risk were calculated by risk exposures, estimates of relative risk, and TMREL, as follow equation:


(1) $$\begin{array}{r} PAF=\frac{P\left(RR-1\right)}{P\left(RR-1\right)+1}\; \end{array}$$

P represents the exposure rate of a certain risk factor level (beyond the TMREL), RR stands for the relative rate of the risk factor. Eventually, the deaths and DALYs attributable to each risk were calculated by multiplying the number of deaths or DALYs for the outcome by the PAF for the risk-outcome pair (1).

### Sociodemographic Index

Socio-demographic Index (SDI) of a certain area was the summary indicator of the development spectrum. SDI was a composite average of the rankings of total fertility rate among females younger than 25 years, educational attainment for those aged 15 years or older, and lag distributed income per capita in the GBD study, and the value ranged (least developed) to 1 (most developed).

### Statistical Analysis

Uncertainty was propagated through all calculations by sampling 1,000 draws at each calculation step. The 2.5th and 97.5th percentiles of sampling 1,000 draws at each calculation step was defined as the 95% uncertainty intervals (UI). For all estimates, a 95% UI excluding zero were considered statistically significant.

## Results

### National Level

**Figure 1:**
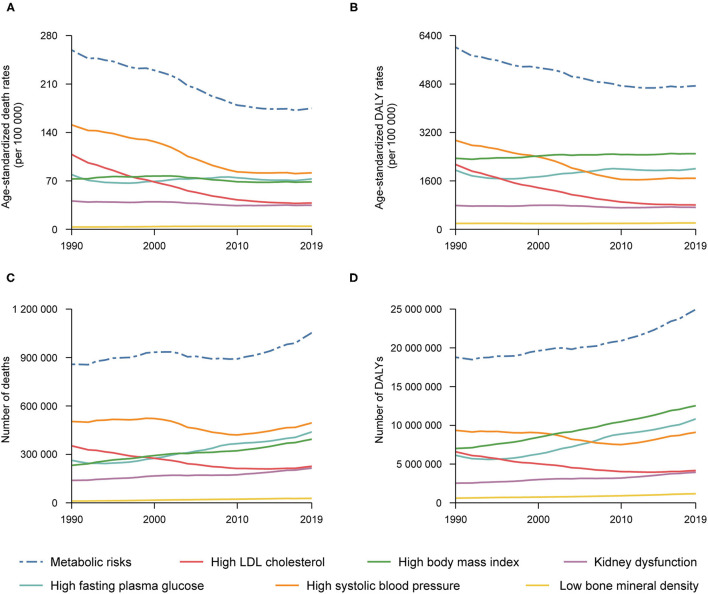
Numbers and age-standardized rates of deaths and DALYs attributable to individual and combined effects of metabolic risks in the United States, 1990–2019: Age-standardized rates of deaths **(A)** and number of deaths **(C)** attributable to individual and combined effects of metabolic risks, and age-standardized DALYs rates **(B)** and number of DALYs **(D)** attributable to individual and combined effects of metabolic risks.

As shown in Figure 1 and Supplementary Table 1, in 2019, the total number of deaths and DALYs attributable to metabolic risks within the US increased to 1,053,576 (95% UI 38,617 to 1,166,534) and 24,951,913 (95% UI 21,921,514 to 28,238,788) years from 1990. The age-standardized death and DALY rates attributable to metabolic risk factors were 174.9 (95% UI 156.6 to 192.8) and 4738.7 (95% UI 4151.9 to 5371.9) per 100,000 people, accounting for 33.1 and 18.2% of age-standardized death and DALY rates from all causes in the US, and significantly decreased by −32.5% (95% UI −35.4 to −29.2) and −21.2% (95% UI −24.6 to −18.0) from 1990, respectively.

For each individual metabolic risk, in 2019, the age-standardized death rate attributable to high SBP was highest [81.6 (95% UI 68.6 to 94.2) per 100,000 people] (Supplementary Table 4), followed by high FPG and high BMI [72.7 (95% UI 54.2 to 95.4) and 68.5 (95% UI 45.7 to 90.5) per 100,000 people] (Supplementary Tables 2, 5), and the age-standardized DALY rate was also higher for high BMI, high FPG and high SBP [2498.2 (95% UI 1759.9 to 3203.1), 2003.3 (95% UI 1629.3 to 2423.8) and 1684.1 (95% UI 1478.7 to 1880.9) per 100,000 people, respectively] (Supplementary Tables 2, 4, 5). From 1990 to 2019, there was a large decrease in age-standardized death and DALY rates attributable to high SBP (−45.9% and −42.7%) and high LDL cholesterol (−64.9% and −62.4%) (Supplementary Tables 3, 4), while burden for high FPG and high BMI remained unchanged (Supplementary Tables 2, 5). However, the trend for low BMD was upward by 35.0 and 8.7% for death and DALY (Supplementary Table 6).

### Age-Gender Pattern

**Figure 2:**
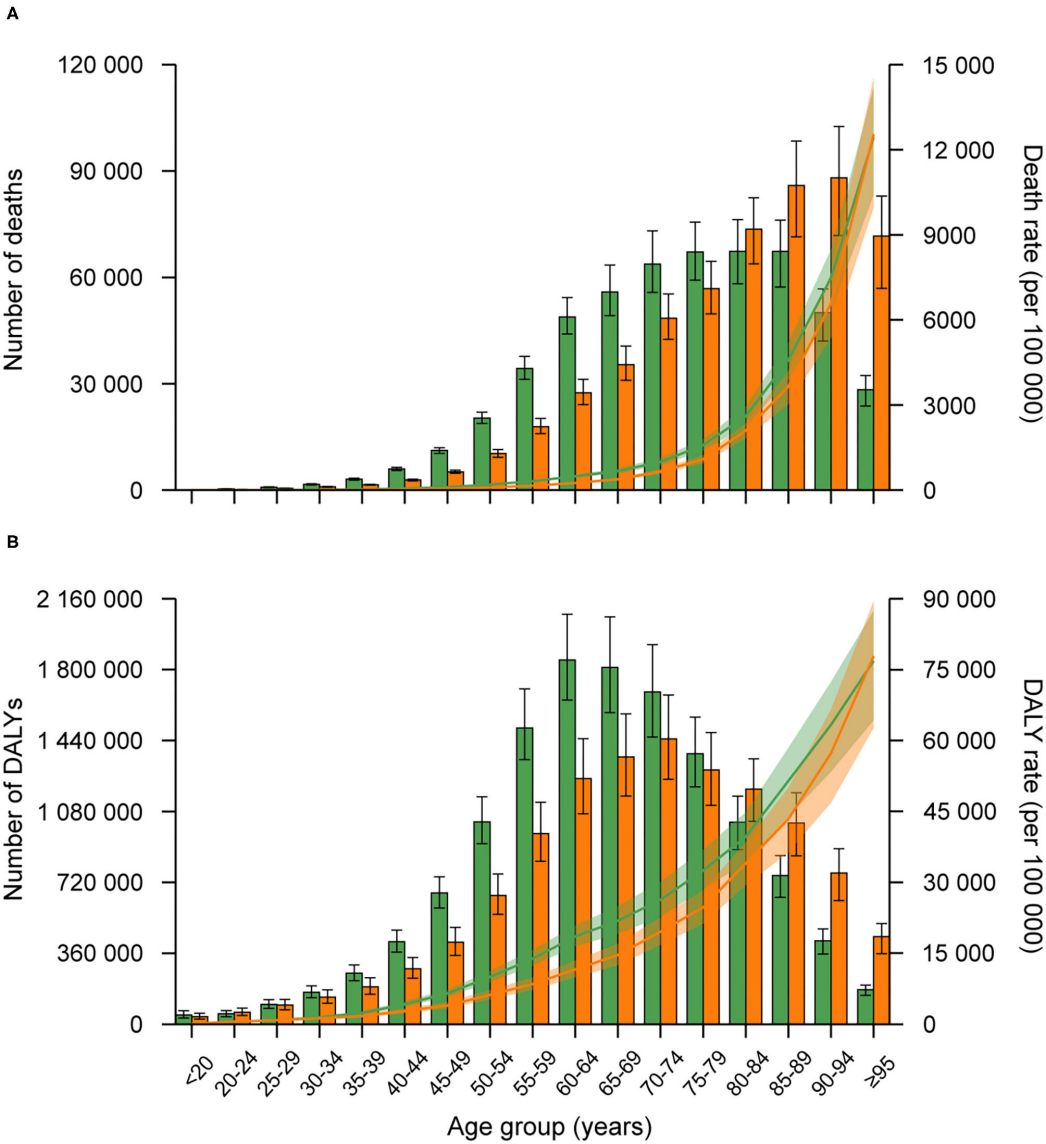
Age-specific numbers and rates of death **(A)**, and DALYs **(B)** attributable to metabolic risk factors by sex, 2019. Error bars indicate the 95% uncertainty interval (UI) for numbers. The green bar indicated males, and orange indicated females.

Generally, the age-specific death and DALY rates attributable to metabolic risks tended to increase with age in both sexes in 2019, and the rates were higher in males than in female across all age groups (although with overlapping uncertainty in some age groups) (Figures 2A,B). The number of deaths attributable to metabolic risks was higher in males than females in age group younger than 75 years, peaking at age group 75–79 years for males and 90–94 for females. For the number of DALYs attributable to metabolic risks, it was higher in males than females in age group younger than 70 years, peaking at age group 60–64 years for males and 90–94 for females.

### By Location

**Figure 3:**
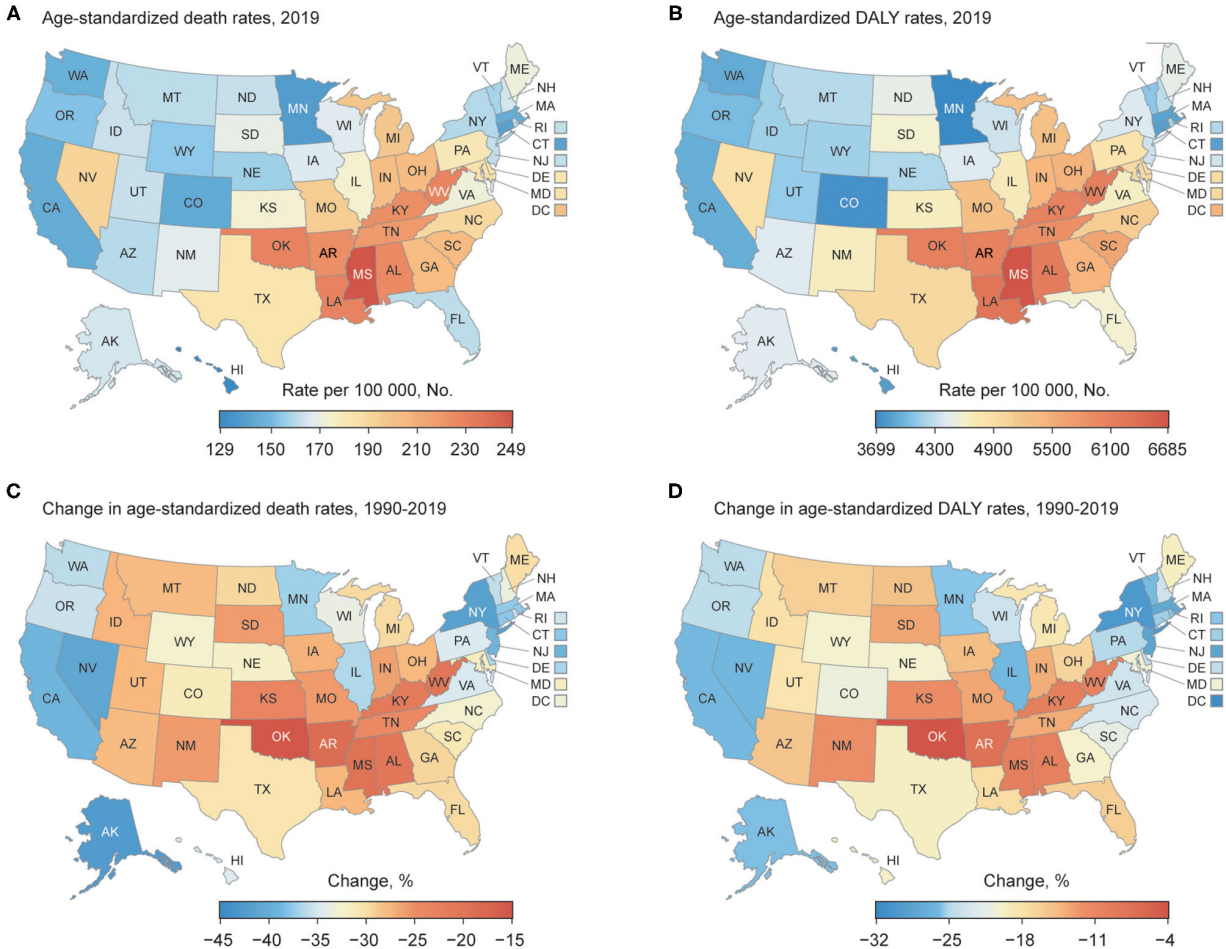
Burden attributable to metabolic risk factors for each state in the US: (**A)** Age-standardized death rate attributable to metabolic risk factors in 2019; **(B)** Age-standardized DALY rate of RIT in 2019; **(C)** Change in age-standardized death rates attributable to metabolic risk factors, 1990–2019; **(D)** Change in age-standardized DALY rates attributable to metabolic risk factors, 1990–2019.

As shown in Figure 3 and Supplementary Tables 1–7, the burden due to metabolic risks varied widely across the country: the highest age-standardized death rate attributable to metabolic risks was mainly seen in states in East and West South Central of the US, such as Mississippi [248.9 (95% UI 210.9 to 293.1)] and Louisiana [229.7 (95% UI 191.6 to 272.6) per 100,000 people], while the lowest was observed in Hawaii [129.4 (95% UI 107.2 to 154.7) per 100,000 people] (Figure 3A). From 1990 to 2019, the greatest improvement was achieved in New York [−41.7% (95% UI −50.4 to −32.9)], but less was in states in East and West South Central [e.g., Oklahoma −15.3% (95% UI −27.7 to −3.0), and Arkansas −18.6% (95% UI −30.8 to −5.9)] (Figure 3C).

The pattern of the age-standardized DALY rate attributable to metabolic risks and its change was analogous to the age-standardized death rate, highest in states in East and West South Central [e.g., Mississippi 6684.9 (95% UI 5671.0 to 7853.3), and Louisiana 6279.9 (95% UI 5243.0 to 7327.8) per 100,000 people], and lowest in Minnesota [3699.7 (95% UI 3085.4 to 4326.2) per 100,000 people] (Figure 3B). As indicated in Figure 3D, the decrease was greatest in District of Columbia [−31.6% (95% UI −40.1 to −22.3)], and least in Oklahoma [−4.1% (95% UI −15.4 to 7.5)]. In addition, the pattern and trend of age-standardized DALY and death rate attributable to individual metabolic risk were very analogous to metabolic risks combined (Supplementary Tables 1–7).

**Figure 4:**
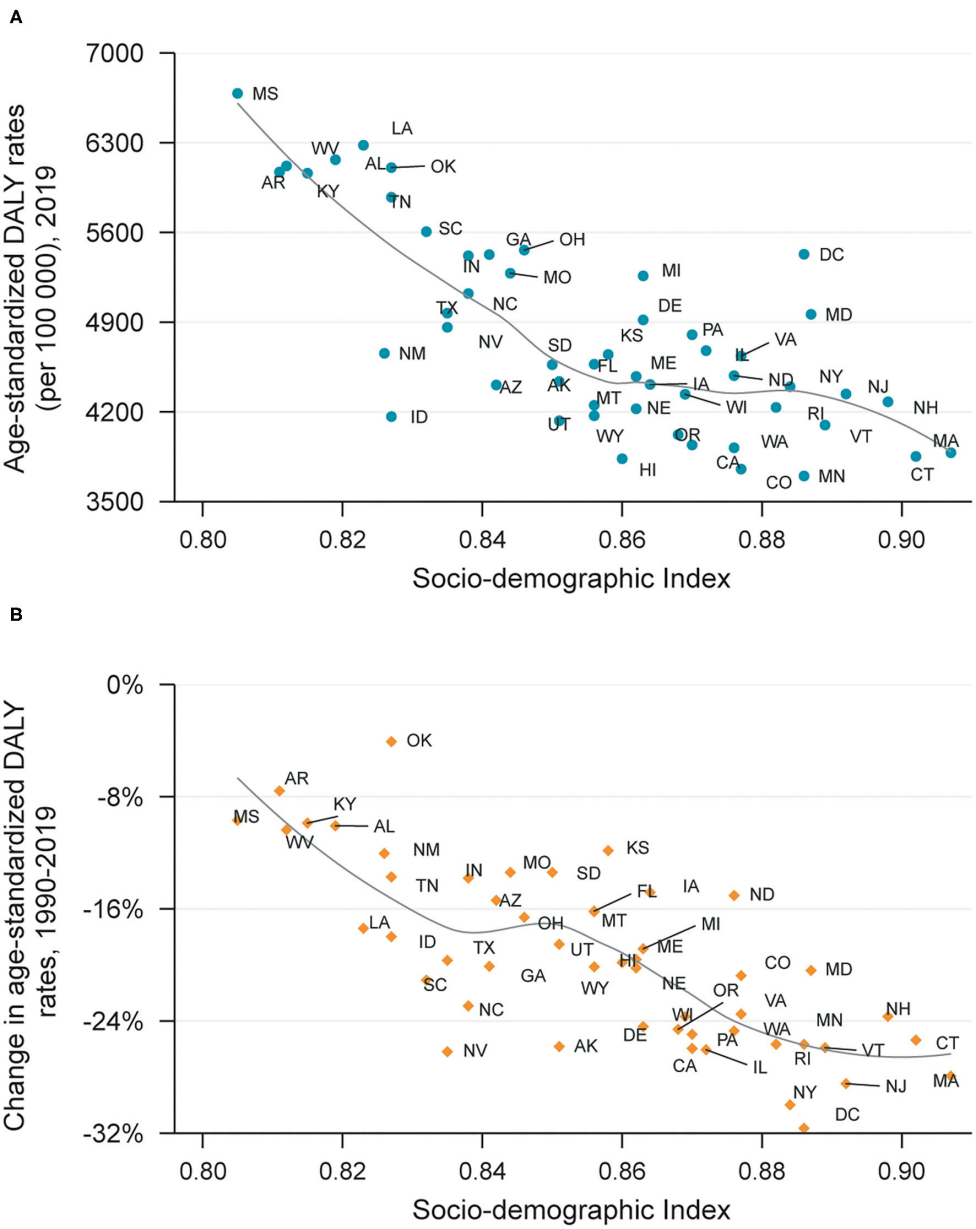
DALYs **(A)** and its changes **(B)** attributable to metabolic risk factors by SDI, 1990–2019.

Generally, the age-standardized DALY rate attributable to metabolic factors and its changes in a certain state was in inverse proportion to its SDI level in 2019 (Figures 4A,B), but some states like Oklahoma had much higher age-standardized DALY rate than expected based on SDI level. Furthermore, states with lower SDI level tended to have minor improvement in DALYs due to metabolic risks, such as Mississippi and Arkansas.

### By Causes

**Figure 5:**
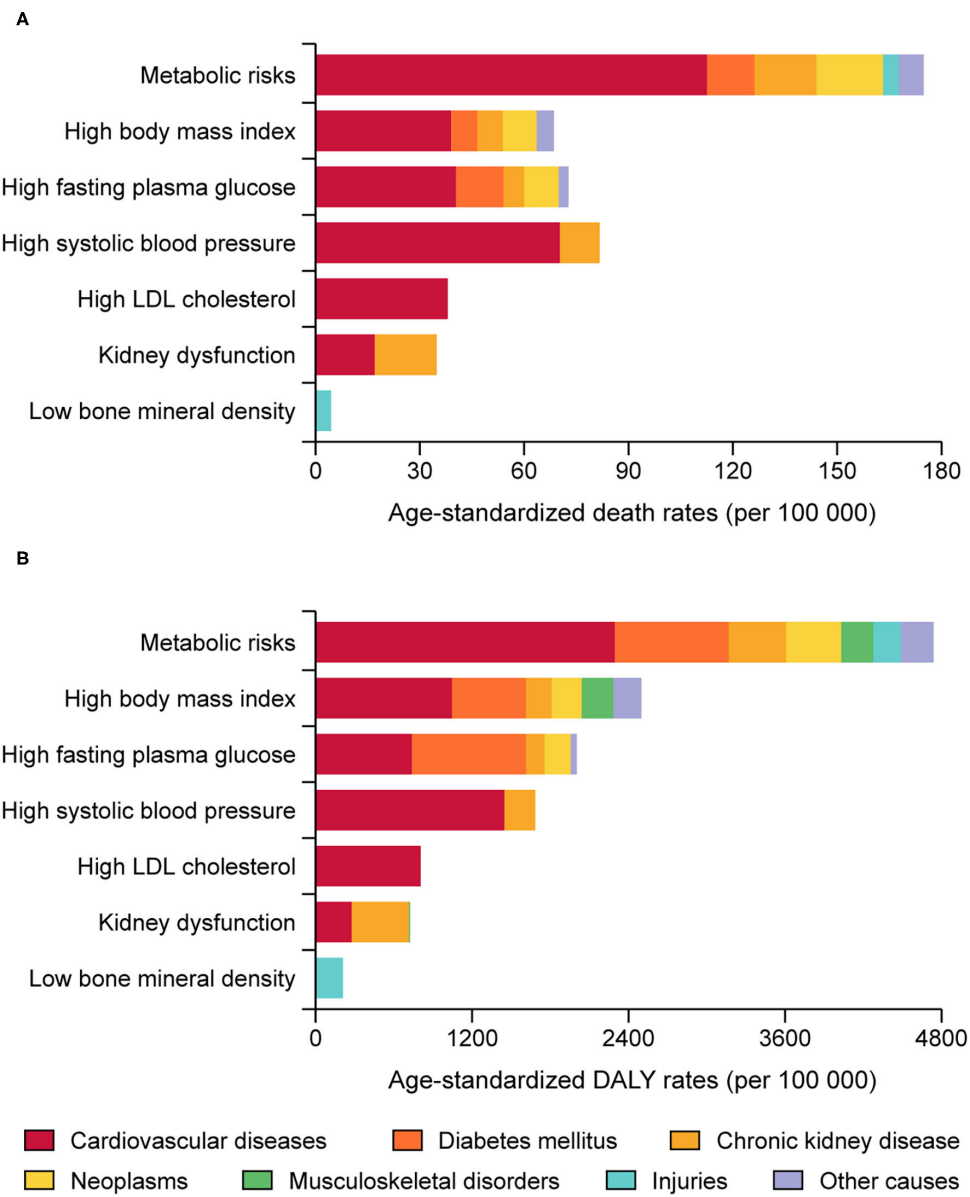
Death **(A)** and DALYs **(B)** attributable to the individual and combined effects of metabolic risks in the United States by diseases.

As shown in Figure 5 and Supplementary Table 8, cardiovascular diseases accounted for the largest proportion of age-standardized death rate attributable to metabolic risks combined (64.4%), followed by neoplasms (11.0%) (Figure 5A). Cardiovascular diseases also took the largest proportion of age-standardized death rate due to each metabolic risk except low BMD. The age-standardized DALY rate attributable to metabolic risks combined, cardiovascular diseases took the largest proportion (48.4%), followed by diabetes mellitus (18.5%) (Figure 5B). In addition, it was indicated that cardiovascular diseases and injuries covered all the DALY rate attributable to high LDL cholesterol and low BMD.

Supplementary Tables 9, 10 provided the age-standardized proportion of deaths and DALYs attributable to the individual and combined effects of metabolic risks. Apart from diabetes mellitus, chronic kidney disease and hypertensive heart disease directly caused by one metabolic risk, the age-standardized proportion of death and DALY attributed to metabolic risks combined was highest for ischemic heart disease (the PAFs were 82.6 and 84.7%, respectively), followed by stroke (the PAFs were 66.1 and 69.5%, respectively). Concerning diabetes mellitus, high BMI attributed high proportion to the age-standardized death and DALY (the PAFs were 55.0 and 64.6%, respectively). Among neoplasms, the age-standardized proportion of death and DALY of uterine cancer was highly affected by high BMI (the PAFs were 56.1 and 54.5%, respectively), followed by esophagus cancer (the PAFs were 34.6 and 36.1%, respectively). While high FPG had large influence on the age-standardized death and DALY of bladder cancer.

## Discussion

Our finding revealed that the number of death and DALY attributable to metabolic risks combined increased remarkably from 1990, however, after standardization of age structure, substantial decreases both in death and DALY were observed, which could be partly explained by the aging of population in the US.

We found out that there was a decline in burden attributable to high SBP and high LDL cholesterol, partly because hypertension control rate increased from 31.6% in 1999 to 48.3% in 2016, despite almost unchanged prevalence of hypertension (20), and a decrease by 0.21 mmol/L per decade in total cholesterol in the US was observed since 1980 (21). While the situation of high BMI and high FPG had not been ameliorated, despite improvement in healthcare. The age-adjusted prevalence of obesity and diabetes in adults increased persistently from 30.5 and 6.4% in 1999, to 42.4 and 9.4% in 2017, respectively (6, 22), and the obesity rate in the US remained 7.7% for a decade (23). Another prominent issue was low BMD. Due to the aging of population, it was projected that the number of adults 50 years and older with low BMD in 2030 would reach to 57.4 million, increased by 32% since 2010 (8).

Our results indicated that the age-standardized death and DALY rate were higher in males than females, but the disparity narrowed and became indistinct after aged 70 years and over, besides, the number of death and DALY attributable to metabolic risks were also higher in female after 70 years old. The reason of the phenomenon is not fully unveiled. There were also some studies reporting that prevalence of high BMI, SBP, and LDL cholesterol were higher in older females than older males in the US (6, 24, 25), and since reduce in estrogen after menopause, females were more susceptible and vulnerable to low BMD (8, 26). Also, this phenomenon was observed in many other countries (27), implying a necessity to understand the sex differences in metabolic risks and integrate them into medical intervention (4, 26, 28).

Through evaluation of burden in each state, we found out wide variations existing in the US, and identified the hotspots hit by metabolic risks. The reasons behind the disparities are multifactorial, including difference in diet habits, demography, ethnicity, healthcare resource and utilization (24), while an important indicator is SDI level (4). However, the effects of SDI were complicated and non-linear, for instance, the age-standardized DALY rate of high BMI increased until SDI is about 0.78, but then decreases with higher SDI which could be explained by the undernutrition in low SDI countries, and much healthier food in high SDI countries (4). The complex relationship between SDI and DALY needed further studied. While our results showed that in the US, the burden attributable to metabolic risks of each state and its change were generally in inverse proportion to local SDI level, which also roughly accorded with the heatmap of metabolic risks distribution in the US, for instance, Mississippi had highest obesity and diabetes rate (40.8 and 12.9%, respectively), also heaviest burden (6), suggesting that prior concerns and interventions should be implemented in locations with low SDI in the US.

As previously reported, cardiovascular diseases (CVD), more specifically, ischemic heart disease and stroke were the leading causes of death and DALY related to metabolic risks in the US (10), and the burden mainly originated from high SBP, LDL cholesterol, BMI, and FPG (25). Last decades had seen the improvements achieved in cardiovascular diseases, which implied the significant role of treatment and prevention of reducing death rate related to metabolic risks (9). With standard statin regimens, a 1.0 mmol/l reduction in LDL resulted in preventing events of heart attack and ischemic stroke by over 20% (29), and apart from wide utilization of statin drugs, replacement of saturated fats with unsaturated fats also leaded to a substantial decrease in burden due to high LDL cholesterol in the US (21). High SBP also had impacts on cardiovascular diseases, and increased the risk of mortality in coronary heart disease (RR = 1.10) and stroke (RR = 1.15) (30), while hypertension control rate increased from 31.6% in 1999 to 48.3% in 2016 (20), thus reducing in cardiovascular risk (31). High FPG, another rising risk, also increased risk for CVD mortality, and the awareness, treatment, and control rate of blood glucose in the US were far from satisfactory (25). Some new anti-diabetic medications like SGLT-2 inhibitor and GLP-1 receptor agonist had been proven to have cardiovascular protection effect. In a meta-analysis study, it was documented that GLP-1 receptor agonist and SGLT-2 inhibitor reduced the risk of CVD mortality (OR = 0.87 and 0.82, respectively), and had potentials in reducing renal dysfunction (32). However, considering that these drugs had not been on the market for a long time, their impacts on CVD are yet to come. Furthermore, some anti-diabetes medications unable to provide cardiovascular protection like sulfonylurea, thiazolidinedione still hold large proportion in the US (33). Concerning high BMI, although an obesity paradox outcome of cardiovascular diseases was reported previously, our study showed that upward in prevalence of high BMI contributed a substantial and increasing burden in cardiovascular diseases, corroborating the results that obesity increased incidence of cardiovascular events in the US (HR = 1.67 for males and 1.85 for females) (34).

Obesity is believed to be a promoter of diabetes mellitus, and high BMI was prevalent in the diabetic patients, which was 27.6% with overweight (BMI 25.0 to 29.9 kg/m^2^), 45.8% with obesity (BMI 30.0 to 39.9 kg/m^2^) and 15.5% with extreme obesity (BMI 40.0 kg/m^2^ or higher) in the US (22). However, the relationship between BMI and all-cause mortality in diabetic patients was controversy, Generally speaking, obesity was deemed as a harmful roles in many diseases including diabetes (35, 36), but many studies indicated reduced risk of all-cause mortality in high BMI diabetic patients (37), which was so called obesity paradox in diabetes. This phenomenon might be explained as follow: patients with high BMI might have higher muscle mass and better health condition (38), moreover, fat tissue is the main energy storage in human body, which could protect them from energy wasting and inflammation, also, fat cell could secrete some cytokines, like adiponectin and leptin, and protect the human body from hyperglycemia (39). While a meta-analysis indicated that obesity paradox might be originated from epidemiological bias and limitation of BMI, and the results showed that mortality risk was lower in patients with BMI interval 25.0 to 30.0 kg/m^2^, but higher in BMI higher than 30.0 kg/m^2^ (36). Our results from GBD data indicated that age-standardized proportion of DALYs and deaths in the US were attributable to high BMI. Considering large proportion of BMI higher than 30.0 kg/m^2^ in the US, efforts on healthy eating and active living are still of importance in US residents.

There are abundant evidences suggesting that cancer incidence and mortality were associated with obesity and diabetes (40–42). In 2012, 5.7% of all incident cancers (804 100 cases) were attributable to the combined effects of diabetes and high BMI (293 300 cases and 544 300 cases, respectively) (42), and the number is undoubtedly increasing with epidemic of obesity and diabetes worldwide. High BMI could increase incidence risk for most sites of cancers, especially for uterine cancer, esophageal cancer, and kidney cancer (RR = 1.50, 1.48 and 1.30 for every 5 kg/m^2^ increase in BMI) (41). Diabetes also increased incidence and mortality of many cancers of high prevalent in the US like breast, lung and colorectal cancer (40, 43). Diabetes and high BMI combined was associated with more than 7% of all incidence cancers (42), calling the need for preventing and controlling excess body weight and blood glucose.

Our work provided a detailed information on burden attributable to metabolic risks combined and individual, and depicted it from different dimensions. However, our results are unavoidably affected by some limitations and uncertainties. First, our data included self-report dataset thus resulting in measurement uncertainty. Second, limitations exist in index of metabolic risks, for instance, although BMI is a popular metric for obesity, it was not a very accuracy parameter because it is not able to account for bone density and body composition, besides, some metabolic risks like hyperuricemia, electrolyte disorder and hormone disturbance which might be related to health loss and death are not incorporated in GBD studies. Third, the burden attributable to metabolic risks varied in ethnicity, rural and urban, and customs, which could not be distinguished by our study.

In conclusion, metabolic risks are important contributor to health burden in the US, and impact mainly on cardiovascular diseases. In addition, through analysis by age-gender, location, high-risk population and hotspot states hit by metabolic risks are identified, providing information and evidence for health workers to attach close concerns, and allocate specific strategies to reduce burden attributable to metabolic risks.

## Data Availability Statement

Publicly available datasets were analyzed in this study. This data can be found here: http://ghdx.healthdata.org/gbd-2019.

## Author Contributions

RC: formal analysis, funding acquisition, and investigation. SS: conceptualization, methodology, and supervision. MB: data curation, formal analysis, and visualization. JK: visualization and investigation. MZ and WZha: methodology and investigation. NB: investigation, methodology, and software. WZho: conceptualization, methodology, supervision, and investigation. All authors contributed to writing and revision of this manuscript and approved the final version of the submitted manuscript.

## Funding

The GBD (Global Burden of Disease) 2019 study was supported by the Bill and Melinda Gates Foundation, and conducted by Institute for Health Metrics and Evaluation (IHME). The present study based on GBD 2019 was funded by the Hunan Provincial Natural Science Foundation (grant number 2019JJ50908).

## Conflict of Interest

The authors declare that the research was conducted in the absence of any commercial or financial relationships that could be construed as a potential conflict of interest.

## Publisher's Note

All claims expressed in this article are solely those of the authors and do not necessarily represent those of their affiliated organizations, or those of the publisher, the editors and the reviewers. Any product that may be evaluated in this article, or claim that may be made by its manufacturer, is not guaranteed or endorsed by the publisher.

## References

[1] Collaborators GBDRF. Global burden of 87 risk factors in 204 countries and territories, 1990-2019: a systematic analysis for the Global Burden of Disease Study 2019. Lancet. (2020) 396:1223–49. 10.1016/S0140-6736(20)30752-2 33069327 PMC7566194

[2] Kochanek KD Xu J Arias E. Mortality in the United States, 2019. NCHS Data Brief. (2019) 395:1–8. 33395387

[3] Ye L Xu J Zhang T Lin X Pan X Zeng W. Global burden of noncommunicable diseases attributable to high fasting plasma glucose. J Diabetes. (2020) 12:807–18. 10.1111/1753-0407.13072 32472661

[4] Dai H Alsalhe TA Chalghaf N Ricco M Bragazzi NL Wu J. The global burden of disease attributable to high body mass index in 195 countries and territories, 1990-2017: an analysis of the Global Burden of Disease Study. PLoS Med. (2020) 17:e1003198. 10.1371/journal.pmed.1003198 32722671 PMC7386577

[5] Li Y Pan A Wang DD Liu X Dhana K Franco OH. Impact of healthy lifestyle factors on life expectancies in the US population. Circulation. (2018) 138:345–55. 10.1161/CIRCULATIONAHA.117.032047 29712712 PMC6207481

[6] Fryar CD Carroll MD Ansai N Lipphardt A. Prevalence of Selected Measures Among Adults Aged 20 and Over: United States, 1999–2000 Through 2017–2018. National Center for Health Statistics (2020).

[7] Vart P Powe NR McCulloch CE Saran R Gillespie BW Saydah S. National trends in the prevalence of chronic kidney disease among racial/ethnic and socioeconomic status groups, 1988-2016. JAMA Netw Open. (2020) 3:e207932. 10.1001/jamanetworkopen.2020.7932 32672828 PMC7366187

[8] Wright NC Looker AC Saag KG Curtis JR Delzell ES Randall S. The recent prevalence of osteoporosis and low bone mass in the United States based on bone mineral density at the femoral neck or lumbar spine. J Bone Miner Res. (2014) 29:2520–6. 10.1002/jbmr.2269 24771492 PMC4757905

[9] Collaborators USBoD Mokdad AH Ballestros K Echko M Glenn S Olsen HE. The state of US Health, 1990-2016: burden of diseases, injuries, and risk factors among US States. JAMA. (2018) 319:1444–72. 10.1001/jama.2018.0158 29634829 PMC5933332

[10] Danaei G Ding EL Mozaffarian D Taylor B Rehm J Murray CJ. The preventable causes of death in the United States: comparative risk assessment of dietary, lifestyle, and metabolic risk factors. PLoS Med. (2009) 6:e1000058. 10.1371/journal.pmed.1000058 19399161 PMC2667673

[11] Group NCDRFC-AW. Trends in cardiometabolic risk factors in the Americas between 1980 and 2014: a pooled analysis of population-based surveys. Lancet Glob Health. (2020) 8:e123–33. 10.1016/S2214-109X(19)30484-X 31839128 PMC7025323

[12] Diseases GBD Injuries C. Global burden of 369 diseases and injuries in 204 countries and territories, 1990-2019: a systematic analysis for the Global Burden of Disease Study 2019. Lancet. (2020) 396:1204–22. 10.1016/S0140-6736(20)30925-9 33069326 PMC7567026

[13] Collaborators GBDD. Global age-sex-specific fertility, mortality, healthy life expectancy (HALE), and population estimates in 204 countries and territories, 1950-2019: a comprehensive demographic analysis for the Global Burden of Disease Study 2019. Lancet. (2020) 396:1160–203. 10.1016/S0140-6736(20)30977-6 33069325 PMC7566045

[14] Cole TJ Bellizzi MC Flegal KM Dietz WH. Establishing a standard definition for child overweight and obesity worldwide: international survey. BMJ. (2000) 320:1240–3. 10.1136/bmj.320.7244.1240 10797032 PMC27365

[15] Moradi-Lakeh M Forouzanfar MH El Bcheraoui C Daoud F Afshin A Hanson SW. High fasting plasma glucose, diabetes, and its risk factors in the Eastern Mediterranean Region, 1990-2013: findings from the Global Burden of Disease Study 2013. Diabetes Care. (2017) 40:22–9. 10.2337/dc16-1075 27797926

[16] Singh GM Danaei G Farzadfar F Stevens GA Woodward M Wormser D. The age-specific quantitative effects of metabolic risk factors on cardiovascular diseases and diabetes: a pooled analysis. PLoS ONE. (2013) 8:e65174. 10.1371/journal.pone.0065174 23935815 PMC3728292

[17] Boekholdt SM Hovingh GK Mora S Arsenault BJ Amarenco P Pedersen TR. Very low levels of atherogenic lipoproteins and the risk for cardiovascular events: a meta-analysis of statin trials. J Am Coll Cardiol. (2014) 64:485–94. 10.1016/j.jacc.2014.02.615 25082583 PMC4443441

[18] Forouzanfar MH Liu P Roth GA Ng M Biryukov S Marczak L. Global burden of hypertension and systolic blood pressure of at least 110 to 115 mm Hg, 1990-2015. JAMA. (2017) 317:165–82. 10.1001/jama.2016.19043 28097354

[19] Mortality GBD Causes Causes of Death C. Global, regional, and national age-sex specific all-cause and cause-specific mortality for 240 causes of death, 1990-2013: a systematic analysis for the Global Burden of Disease Study (2013). Lancet. (2015) 385:117–71. 10.1016/S0140-6736(14)61682-2 25530442 PMC4340604

[20] Fryar CD Ostchega Y Hales CM Zhang G Kruszon-Moran D. Hypertension prevalence and control among adults: United States, 2015-2016. NCHS Data Brief. (2017) 289:1–8. 29155682

[21] Farzadfar F Finucane MM Danaei G Pelizzari PM Cowan MJ Paciorek CJ. National, regional, and global trends in serum total cholesterol since 1980: systematic analysis of health examination surveys and epidemiological studies with 321 country-years and 3.0 million participants. Lancet. (2011) 377:578–86. 10.1016/S0140-6736(10)62038-7 21295847

[22] CDC. National Diabetes Statistics Report 2020: Estimates of Diabetes and Its Burden in the United States (2020).

[23] Hales CM Fryar CD Carroll MD Freedman DS Ogden CL. Trends in obesity and severe obesity prevalence in US youth and adults by sex and age, 2007-2008 to 2015-2016. JAMA. (2018) 319:1723–25. 10.1001/jama.2018.3060 29570750 PMC5876828

[24] National Center for Health Statistics. Health, United States, 2019 Report (2019). 33818995

[25] Virani SS Alonso A Benjamin EJ Bittencourt MS Callaway CW Carson AP. Heart disease and stroke statistics-2020 update: a report from the American Heart Association. Circulation. (2020) 141:e139–596. 10.1161/CIR.0000000000000746 31992061

[26] Eastell R O'Neill TW Hofbauer LC Langdahl B Reid IR Gold DT. Postmenopausal osteoporosis. Nat Rev Dis Primers. (2016) 2:16069. 10.1038/nrdp.2016.69 27681935

[27] Collaborators GBDO Afshin A Forouzanfar MH Reitsma MB Sur P Estep K. Health effects of overweight and obesity in 195 countries over 25 years. N Engl J Med. (2017) 377:13–27. 10.1056/NEJMoa1614362 28604169 PMC5477817

[28] Gillis EE Sullivan JC. Sex differences in hypertension: recent advances. Hypertension. (2016) 68:1322–27. 10.1161/HYPERTENSIONAHA.116.06602 27777357 PMC5159215

[29] Cholesterol Treatment Trialists' (CTT) Collaboration Baigent C Blackwell L Emberson J Holland LE Reith C. Efficacy and safety of more intensive lowering of LDL cholesterol: a meta-analysis of data from 170,000 participants in 26 randomised trials. Lancet. (2010) 376:1670–81. 10.1016/S0140-6736(10)61350-5 21067804 PMC2988224

[30] Stevens SL Wood S Koshiaris C Law K Glasziou P Stevens RJ. Blood pressure variability and cardiovascular disease: systematic review and meta-analysis. BMJ. (2016) 354:i4098. 10.1136/bmj.i4098 27511067 PMC4979357

[31] Zhang Y Moran AE. Trends in the prevalence, awareness, treatment, and control of hypertension among young adults in the United States, 1999 to 2014. Hypertension. (2017) 70:736–42. 10.1161/HYPERTENSIONAHA.117.09801 28847890 PMC5657525

[32] Kanie T Mizuno A Takaoka Y Suzuki T Yoneoka D Nishikawa Y. Dipeptidyl peptidase-4 inhibitors, glucagon-like peptide 1 receptor agonists and sodium-glucose co-transporter-2 inhibitors for people with cardiovascular disease: a network meta-analysis. Cochrane Database Syst Rev. (2021) 10:CD013650. 10.1002/14651858.CD013650.pub2 34693515 PMC8812344

[33] Liu L Simon B Shi J Mallhi AK Eisen HJ. Impact of diabetes mellitus on risk of cardiovascular disease and all-cause mortality: evidence on health outcomes and antidiabetic treatment in United States adults. World J Diabetes. (2016) 7:449–61. 10.4239/wjd.v7.i18.449 27795819 PMC5065665

[34] Khan SS Ning H Wilkins JT Allen N Carnethon M Berry JD. Association of body mass index with lifetime risk of cardiovascular disease and compression of morbidity. JAMA Cardiol. (2018) 3:280–7. 10.1001/jamacardio.2018.0022 29490333 PMC5875319

[35] Flegal KM Kit BK Orpana H Graubard BI. Association of all-cause mortality with overweight and obesity using standard body mass index categories: a systematic review and meta-analysis. JAMA. (2013) 309:71–82. 10.1001/jama.2012.113905 23280227 PMC4855514

[36] Kwon Y Kim HJ Park S Park YG Cho KH. Body mass index-related mortality in patients with type 2 diabetes and heterogeneity in obesity paradox studies: a dose-response meta-analysis. PLoS ONE. (2017) 12:e0168247. 10.1371/journal.pone.0168247 28046128 PMC5207428

[37] Liu XM Liu YJ Zhan J He QQ. Overweight, obesity and risk of all-cause and cardiovascular mortality in patients with type 2 diabetes mellitus: a dose-response meta-analysis of prospective cohort studies. Eur J Epidemiol. (2015) 30:35–45. 10.1007/s10654-014-9973-5 25421785

[38] McAuley PA Kokkinos PF Oliveira RB Emerson BT Myers JN. Obesity paradox and cardiorespiratory fitness in 12,417 male veterans aged 40 to 70 years. Mayo Clin Proc. (2010) 85:115–21. 10.4065/mcp.2009.0562 20118386 PMC2813818

[39] Then C Herder C Thorand B Sujana C Heier M Meisinger C. Association of serum uromodulin with adipokines in dependence of type 2 diabetes. Cytokine. (2021) 150:155786. 10.1016/j.cyto.2021.155786 34920231

[40] Giovannucci E Harlan DM Archer MC Bergenstal RM Gapstur SM Habel LA. Diabetes and cancer: a consensus report. CA Cancer J Clin. (2010) 60:207–21. 10.3322/caac.20078 20554718

[41] Sung H Siegel RL Torre LA Pearson-Stuttard J Islami F Fedewa SA. Global patterns in excess body weight and the associated cancer burden. CA Cancer J Clin. (2019) 69:88–112. 10.3322/caac.21499 30548482

[42] Pearson-Stuttard J Zhou B Kontis V Bentham J Gunter MJ Ezzati M. Worldwide burden of cancer attributable to diabetes and high body-mass index: a comparative risk assessment. Lancet Diabetes Endocrinol. (2018) 6:e6–15. 10.1016/S2213-8587(18)30150-5 29803268 PMC5982644

[43] Siegel RL Miller KD Fuchs HE Jemal A. Cancer statistics, 2021. CA Cancer J Clin. (2021) 71:7–33. 10.3322/caac.21654 33433946

