# Supplementary material for: Health Effects of Metabolic Risks in the United States From 1990 to 2019
Source: Front Public Health. 2022 Jan 31;10:751126. doi: 10.3389/fpubh.2022.751126 (PMC8841675; doi:10.3389/fpubh.2022.751126)
Supplement: Supplementary file 1 [file Data_Sheet_1.docx]

**Supplement file for review**

**Table S1.** Deaths and DALYs attributable to metabolic risks in 2019 and percentage change of age-standardized rates from 1990 to 2019, by location.

**Table S2**. Deaths and DALYs attributable to high fasting plasma glucose in 2019 and percentage change of age-standardised rates from 1990 to 2019, by location.

**Table S3.** Deaths and DALYs attributable to high LDL cholesterol in 2019 and percentage change of age-standardised rates from 1990 to 2019, by location.

**Table S4**. Deaths and DALYs attributable to high systolic blood pressure in 2019 and percentage change of age-standardised rates from 1990 to 2019, by location.

**Table S5.** Deaths and DALYs attributable to high body mass index in 2019 and percentage change of age-standardised rates from 1990 to 2019, by location.

**Table S6.** Deaths and DALYs attributable to low bone mineral density in 2019 and percentage change of age-standardised rates from 1990 to 2019, by location.

**Table S7.** Deaths and DALYs attributable to kidney dysfunction in 2019 and percentage change of age-standardised rates from 1990 to 2019, by location.

**Table S8.** Age-standardized death and DALYs rate attributable to the individual and combined effects of metabolic risks in the United States by diseases.

**Table S9.** Age-standardised proportion of deaths attributable to the individual and combined effects of metabolic risks in the United States, 2019.

**Table S10.** Age-standardised proportion of DALYs attributable to the individual and combined effects of metabolic risks in the United States, 2019.

**Table S1**. Deaths and DALYs attributable to metabolic risks in 2019 and percentage change of age-standardised rates from 1990 to 2019, by location.

|  | Deaths |  |  | DALYs |  |  |
| --- | --- | --- | --- | --- | --- | --- |
|  | Counts | Age-standardised rates per 100,000 people | Percentage change in age-standardised rates, 1990-2019 | Counts | Age-standardised rates per 100,000 people | Percentage change in age-standardised rates, 1990-2019 |
| United States | 1053576 (938617 to 1166534) | 174.9 (156.6 to 192.8) | -32.5% (-35.4 to -29.2) | 24951913 (21921514 to 28238788) | 4738.7 (4151.9 to 5371.9) | -21.2% (-24.6 to -18.0) |
| Alabama | 19953 (16722 to 23664) | 226.6 (190.6 to 269.3) | -20.5% (-31.2 to -8.0) | 488077 (410116 to 569114) | 6167.1 (5177.4 to 7181.8) | -10.1% (-20.2 to 1.7) |
| Alaska | 1583 (1347 to 1835) | 164.2 (139.7 to 189.3) | -42.6% (-49.5 to -35.0) | 45411 (38190 to 52750) | 4437.0 (3745.5 to 5147.8) | -25.8% (-33.2 to -17.9) |
| Arizona | 21847 (18144 to 25855) | 159.8 (132.0 to 190.0) | -27.8% (-37.9 to -16.2) | 517581 (433794 to 612262) | 4409.5 (3690.9 to 5241.6) | -15.4% (-24.6 to -4.9) |
| Arkansas | 12452 (10423 to 14650) | 224.4 (188.0 to 264.7) | -18.6% (-30.8 to -5.9) | 294370 (248589 to 343392) | 6071.4 (5111.0 to 7092.1) | -7.6% (-19.2 to 5.0) |
| California | 100497 (83667 to 117853) | 144.5 (119.7 to 170.2) | -39.0% (-47.6 to -29.8) | 2381566 (1999338 to 2789191) | 3941.4 (3312.1 to 4640.7) | -26.0% (-34.2 to -17.3) |
| Colorado | 12554 (10419 to 14878) | 143.5 (118.9 to 170.1) | -31.1% (-41.2 to -20.4) | 309060 (258239 to 364423) | 3753.1 (3125.6 to 4440.1) | -20.8% (-30.2 to -11.4) |
| Connecticut | 11188 (9142 to 13602) | 140.0 (113.6 to 171.1) | -37.7% (-48.0 to -27.3) | 252195 (207919 to 301198) | 3852.2 (3178.7 to 4604.5) | -25.4% (-34.3 to -16.7) |
| Delaware | 3459 (2941 to 4025) | 181.6 (153.9 to 211.4) | -36.8% (-44.5 to -28.6) | 82365 (69934 to 94991) | 4917.5 (4164.2 to 5684.2) | -24.4% (-32.2 to -16.6) |
| District of Columbia | 2023 (1695 to 2377) | 200.2 (167.6 to 237.2) | -32.8% (-42.2 to -22.4) | 48091 (40793 to 56303) | 5429.5 (4585.6 to 6376.0) | -31.6% (-40.1 to -22.3) |
| Florida | 79684 (66133 to 94633) | 160.7 (132.6 to 192.1) | -29.7% (-40.3 to -18.3) | 1795988 (1508741 to 2106950) | 4571.4 (3818.8 to 5375.2) | -16.2% (-26.7 to -5.8) |
| Georgia | 31481 (26170 to 37604) | 203.2 (169.2 to 242.2) | -29.4% (-39.4 to -18.3) | 826089 (702680 to 976912) | 5426.9 (4615.4 to 6406.2) | -20.1% (-29.6 to -9.5) |
| Hawaii | 4245 (3539 to 5040) | 129.4 (107.2 to 154.7) | -34.7% (-44.2 to -24.1) | 99441 (82823 to 117608) | 3834.1 (3188.1 to 4561.5) | -19.8% (-29.1 to -9.5) |
| Idaho | 4708 (3984 to 5530) | 162.4 (137.7 to 190.7) | -27.4% (-37.6 to -15.7) | 110424 (94266 to 130344) | 4162.9 (3543.5 to 4916.5) | -18.0% (-27.0 to -8.0) |
| Illinois | 41574 (35041 to 48807) | 173.6 (145.3 to 204.9) | -36.5% (-45.7 to -26.3) | 970644 (822368 to 1134975) | 4677.9 (3945.5 to 5500.7) | -26.0% (-34.8 to -16.5) |
| Indiana | 24054 (19875 to 28574) | 202.1 (166.5 to 239.6) | -26.1% (-36.8 to -14.0) | 573130 (482920 to 671662) | 5417.9 (4563.3 to 6377.1) | -13.8% (-23.8 to -2.9) |
| Iowa | 11074 (9296 to 13092) | 165.8 (137.7 to 197.4) | -27.2% (-38.5 to -15.3) | 237690 (199247 to 281473) | 4413.6 (3664.3 to 5245.7) | -14.8% (-25.2 to -3.3) |
| Kansas | 9485 (7885 to 11251) | 173.0 (143.4 to 206.3) | -23.2% (-34.8 to -10.7) | 217267 (181882 to 256768) | 4647.6 (3875.4 to 5497.4) | -11.8% (-22.3 to -0.4) |
| Kentucky | 17664 (14720 to 21079) | 224.2 (187.1 to 267.7) | -21.2% (-32.6 to -8.2) | 435330 (366090 to 516323) | 6061.7 (5084.2 to 7186.6) | -9.9% (-20.5 to 1.9) |
| Louisiana | 17646 (14775 to 20923) | 229.7 (191.6 to 272.6) | -27.5% (-38.1 to -16.5) | 437571 (366772 to 510802) | 6279.9 (5243.0 to 7327.8) | -17.4% (-28.0 to -7.3) |
| Maine | 5190 (4328 to 6130) | 171.4 (143.1 to 202.9) | -29.9% (-38.9 to -19.0) | 117776 (99073 to 138621) | 4475.7 (3748.3 to 5292.1) | -19.6% (-28.3 to -9.8) |
| Maryland | 19748 (16224 to 23313) | 184.4 (151.6 to 218.7) | -31.6% (-41.7 to -20.8) | 478650 (399271 to 558719) | 4960.8 (4141.7 to 5782.1) | -20.4% (-29.7 to -10.3) |
| Massachusetts | 20098 (16478 to 23930) | 145.3 (118.8 to 174.2) | -37.5% (-46.3 to -28.4) | 449438 (372941 to 531912) | 3881.1 (3214.7 to 4610.2) | -27.9% (-36.4 to -19.8) |
| Michigan | 38398 (32018 to 45205) | 197.1 (162.9 to 234.2) | -29.7% (-39.8 to -18.7) | 886396 (747959 to 1046078) | 5260.8 (4449.4 to 6223.7) | -18.8% (-28.6 to -9.2) |
| Minnesota | 14707 (12059 to 17630) | 137.8 (112.8 to 165.3) | -37.1% (-46.6 to -26.7) | 338956 (282331 to 395525) | 3699.7 (3085.4 to 4326.2) | -25.7% (-34.1 to -17.2) |
| Mississippi | 12589 (10678 to 14809) | 248.9 (210.9 to 293.1) | -19.5% (-31.0 to -7.3) | 307346 (261182 to 362008) | 6684.9 (5671.0 to 7853.3) | -9.7% (-20.3 to 2.2) |
| Missouri | 23118 (19111 to 27371) | 195.2 (162.0 to 231.8) | -25.7% (-36.8 to -13.5) | 541873 (454288 to 636501) | 5281.2 (4419.5 to 6187.5) | -13.4% (-23.8 to -2.0) |
| Montana | 3437 (2859 to 4054) | 160.6 (133.6 to 189.3) | -27.8% (-37.7 to -17.0) | 79191 (66370 to 93035) | 4251.0 (3560.8 to 4998.3) | -16.2% (-25.7 to -6.1) |
| Nebraska | 5739 (4783 to 6727) | 156.3 (129.4 to 184.0) | -32.0% (-42.1 to -21.9) | 129592 (109543 to 152220) | 4223.8 (3553.5 to 5000.0) | -20.2% (-29.5 to -11.1) |
| Nevada | 9094 (7652 to 10803) | 192.1 (162.0 to 227.7) | -41.0% (-48.9 to -31.4) | 229083 (192482 to 268564) | 4859.5 (4081.4 to 5696.9) | -26.2% (-34.7 to -16.6) |
| New Hampshire | 4442 (3683 to 5310) | 164.2 (136.0 to 196.2) | -33.4% (-43.5 to -22.5) | 104480 (87695 to 123239) | 4278.4 (3599.7 to 5070.5) | -23.7% (-32.4 to -14.1) |
| New Jersey | 29343 (24257 to 35046) | 161.7 (133.4 to 194.1) | -39.0% (-47.8 to -28.4) | 661173 (558031 to 780353) | 4339.5 (3639.6 to 5136.4) | -28.5% (-36.3 to -19.3) |
| New Mexico | 6950 (5882 to 8146) | 167.6 (141.0 to 197.5) | -25.7% (-35.6 to -13.9) | 167173 (141084 to 196877) | 4656.7 (3916.8 to 5512.5) | -12.0% (-21.6 to -1.5) |
| New York | 64550 (53549 to 76237) | 159.2 (131.2 to 189.4) | -41.7% (-50.4 to -32.9) | 1460956 (1229724 to 1719172) | 4396.4 (3697.6 to 5167.7) | -30.0% (-38.2 to -21.8) |
| North Carolina | 34512 (28720 to 40821) | 190.0 (158.3 to 224.8) | -32.6% (-41.9 to -21.7) | 856082 (730321 to 1003311) | 5122.8 (4372.1 to 6011.3) | -22.9% (-31.4 to -12.5) |
| North Dakota | 2406 (2024 to 2781) | 162.1 (136.7 to 188.2) | -29.5% (-38.0 to -19.8) | 52821 (44755 to 61126) | 4482.2 (3774.1 to 5181.9) | -15.0% (-23.5 to -5.5) |
| Ohio | 46419 (39400 to 54648) | 203.8 (173.3 to 239.7) | -27.7% (-36.9 to -17.2) | 1075539 (921866 to 1251728) | 5461.8 (4678.5 to 6361.1) | -16.6% (-25.4 to -6.9) |
| Oklahoma | 15693 (13049 to 18534) | 229.5 (191.0 to 272.0) | -15.3% (-27.7 to -3.0) | 371761 (312996 to 434336) | 6105.2 (5133.5 to 7160.1) | -4.1% (-15.4 to 7.5) |
| Oregon | 12266 (10266 to 14664) | 152.2 (127.2 to 182.6) | -35.6% (-45.0 to -25.5) | 283585 (237827 to 334749) | 4021.6 (3348.4 to 4755.5) | -24.6% (-33.3 to -15.3) |
| Pennsylvania | 50645 (41934 to 60305) | 179.5 (147.3 to 214.2) | -34.8% (-44.0 to -24.1) | 1116800 (936770 to 1315513) | 4801.8 (4006.4 to 5686.9) | -24.9% (-33.9 to -15.7) |
| Rhode Island | 3675 (3055 to 4294) | 161.6 (135.1 to 190.3) | -35.3% (-44.2 to -25.7) | 79073 (66057 to 92031) | 4234.7 (3512.7 to 4967.7) | -25.7% (-34.6 to -17.2) |
| South Carolina | 18183 (15042 to 21603) | 204.1 (168.7 to 243.0) | -30.8% (-40.9 to -19.5) | 457143 (387469 to 535620) | 5606.1 (4732.6 to 6584.6) | -21.1% (-30.5 to -11.2) |
| South Dakota | 2976 (2521 to 3420) | 169.2 (143.3 to 195.7) | -25.8% (-34.8 to -14.9) | 65429 (55797 to 76079) | 4567.9 (3875.0 to 5365.3) | -13.4% (-21.9 to -3.3) |
| Tennessee | 25920 (21566 to 31233) | 219.3 (183.1 to 264.8) | -24.3% (-34.6 to -12.3) | 633212 (532853 to 755245) | 5874.4 (4945.1 to 6986.8) | -13.7% (-23.3 to -2.4) |
| Texas | 73777 (60975 to 86363) | 182.5 (150.9 to 213.9) | -30.5% (-40.4 to -20.2) | 1915665 (1606814 to 2261271) | 4970.0 (4157.7 to 5876.8) | -19.7% (-28.6 to -10.7) |
| Utah | 6250 (5251 to 7391) | 162.3 (136.5 to 192.2) | -27.6% (-37.4 to -16.8) | 154095 (129170 to 182460) | 4130.6 (3461.1 to 4896.5) | -18.5% (-27.5 to -8.7) |
| Vermont | 2179 (1866 to 2498) | 157.5 (134.8 to 181.5) | -35.9% (-43.0 to -27.8) | 48799 (42003 to 56485) | 4096.8 (3515.7 to 4749.4) | -25.9% (-32.7 to -18.2) |
| Virginia | 25074 (20645 to 29566) | 171.7 (140.8 to 202.2) | -34.9% (-44.7 to -25.0) | 624367 (526635 to 729614) | 4635.5 (3900.3 to 5439.7) | -23.5% (-32.5 to -14.5) |
| Washington | 19131 (15799 to 22798) | 146.9 (121.2 to 175.9) | -36.1% (-45.0 to -26.6) | 461206 (382659 to 550725) | 3919.5 (3253.2 to 4688.1) | -24.7% (-33.4 to -15.7) |
| West Virginia | 8994 (7467 to 10711) | 229.1 (190.0 to 273.8) | -20.2% (-31.4 to -7.5) | 208553 (175493 to 245707) | 6118.5 (5154.4 to 7185.5) | -10.4% (-20.7 to 1.1) |
| Wisconsin | 19250 (16066 to 22832) | 166.7 (138.7 to 198.8) | -33.7% (-43.8 to -23.1) | 433284 (363919 to 513661) | 4337.0 (3631.5 to 5133.5) | -23.7% (-32.4 to -14.4) |
| Wyoming | 1649 (1420 to 1903) | 153.8 (132.9 to 177.8) | -32.5% (-39.4 to -24.1) | 40128 (34444 to 46586) | 4169.7 (3570.9 to 4843.3) | -20.1% (-26.9 to -12.3) |

Data in parentheses are 95% uncertainty intervals. DALYs= disability-adjusted life-years.

**Table S2**. Deaths and DALYs attributable to high fasting plasma glucose in 2019 and percentage change of age-standardised rates from 1990 to 2019, by location.

|  | Deaths |  |  | DALYs |  |  |
| --- | --- | --- | --- | --- | --- | --- |
|  | Counts | Age-standardised rates per 100,000 people | Percentage change in age-standardised rates, 1990-2019 | Counts | Age-standardised rates per 100,000 people | Percentage change in age-standardised rates, 1990-2019 |
| United States | 439379 (320109 to 582663) | 72.7 (54.2 to 95.4) | -8.0% (-17.9 to 2.1) | 10827167 (8720976 to 13255259) | 2003.3 (1629.3 to 2423.8) | 2.6% (-6.5 to 11.3) |
| Alabama | 8653 (6293 to 11793) | 97.0 (70.8 to 131.2) | 4.2% (-16.4 to 30.7) | 218351 (173487 to 272586) | 2665.8 (2128.3 to 3294.4) | 13.9% (-3.8 to 35.9) |
| Alaska | 666 (483 to 886) | 68.2 (48.9 to 90.9) | -20.3% (-36.3 to -2.4) | 19389 (15187 to 24418) | 1844.3 (1438.9 to 2302.7) | -3.5% (-18.4 to 12.5) |
| Arizona | 9046 (6445 to 12489) | 66.2 (47.9 to 90.2) | 2.5% (-18.2 to 26.7) | 226188 (176925 to 286231) | 1881.3 (1481.6 to 2369.2) | 13.9% (-4.0 to 33.2) |
| Arkansas | 5275 (3813 to 7203) | 94.1 (68.4 to 126.3) | 13.7% (-10.1 to 41.5) | 128509 (100028 to 161610) | 2563.7 (2004.3 to 3201.9) | 23.2% (2.4 to 46.2) |
| California | 39791 (27719 to 55007) | 57.7 (40.6 to 79.5) | -6.5% (-25.7 to 14.5) | 991560 (763048 to 1247173) | 1613.6 (1244.6 to 2016.0) | 5.9% (-9.7 to 23.4) |
| Colorado | 4708 (3282 to 6601) | 53.5 (37.3 to 74.9) | -5.2% (-25.0 to 19.1) | 120385 (93058 to 153623) | 1425.9 (1112.6 to 1804.9) | 2.6% (-13.7 to 21.1) |
| Connecticut | 4514 (3063 to 6416) | 57.1 (39.2 to 79.9) | -8.9% (-28.6 to 13.7) | 107534 (82537 to 137744) | 1608.2 (1245.6 to 2034.1) | 3.2% (-13.6 to 21.3) |
| Delaware | 1508 (1063 to 2052) | 78.3 (55.7 to 105.6) | -18.0% (-33.1 to -0.5) | 37091 (28903 to 46294) | 2140.6 (1682.5 to 2654.1) | -5.6% (-19.7 to 10.4) |
| District of Columbia | 756 (528 to 1042) | 75.6 (53.9 to 101.7) | -21.5% (-35.8 to -2.5) | 18490 (14497 to 23140) | 2073.0 (1642.0 to 2588.3) | -21.9% (-33.5 to -7.6) |
| Florida | 33433 (22960 to 46571) | 67.4 (47.9 to 92.3) | 3.2% (-18.0 to 26.8) | 790565 (602986 to 1006435) | 1952.5 (1517.3 to 2457.4) | 16.0% (-1.5 to 34.2) |
| Georgia | 13197 (9600 to 17904) | 84.1 (60.9 to 114.1) | -2.5% (-22.8 to 22.0) | 359187 (281204 to 450505) | 2299.0 (1806.6 to 2866.8) | 6.5% (-12.0 to 26.7) |
| Hawaii | 1692 (1188 to 2369) | 52.0 (37.1 to 71.6) | -2.6% (-22.7 to 19.1) | 41857 (32640 to 53377) | 1566.5 (1233.0 to 1991.3) | 11.7% (-5.2 to 28.6) |
| Idaho | 1973 (1404 to 2677) | 67.2 (48.1 to 90.6) | 2.2% (-18.3 to 26.7) | 48058 (37683 to 60107) | 1759.2 (1392.1 to 2183.5) | 9.2% (-7.6 to 27.8) |
| Illinois | 16850 (11891 to 23318) | 70.5 (50.6 to 97.7) | -13.1% (-32.1 to 7.0) | 409617 (315926 to 518152) | 1928.8 (1510.1 to 2417.2) | -3.5% (-19.7 to 12.7) |
| Indiana | 10458 (7383 to 14215) | 87.3 (62.3 to 119.3) | -1.5% (-21.9 to 23.6) | 258313 (203218 to 321358) | 2373.7 (1881.3 to 2934.9) | 10.7% (-7.2 to 31.0) |
| Iowa | 4373 (3002 to 6228) | 66.1 (46.6 to 92.1) | 6.2% (-16.7 to 32.8) | 99035 (76440 to 128751) | 1796.8 (1402.8 to 2309.3) | 17.5% (-1.9 to 38.9) |
| Kansas | 3999 (2792 to 5530) | 73.0 (51.7 to 100.3) | 9.0% (-13.2 to 35.1) | 95312 (74853 to 121344) | 1988.0 (1564.2 to 2524.8) | 17.6% (-1.2 to 36.9) |
| Kentucky | 7733 (5554 to 10409) | 96.9 (70.1 to 129.7) | 4.3% (-18.5 to 31.4) | 195124 (151016 to 245615) | 2629.9 (2054.8 to 3309.5) | 15.0% (-5.3 to 38.4) |
| Louisiana | 7429 (5361 to 10018) | 95.6 (69.5 to 128.8) | -15.4% (-31.9 to 7.1) | 190956 (148704 to 242386) | 2657.0 (2081.3 to 3352.1) | -5.1% (-20.4 to 13.7) |
| Maine | 2219 (1556 to 3077) | 72.7 (51.6 to 100.6) | -2.4% (-21.6 to 21.9) | 52775 (40929 to 67135) | 1932.5 (1515.3 to 2434.6) | 6.4% (-11.3 to 26.7) |
| Maryland | 8222 (5856 to 11303) | 76.3 (54.9 to 104.1) | -12.0% (-29.1 to 9.1) | 204387 (157684 to 253166) | 2062.8 (1605.4 to 2543.6) | -2.7% (-17.7 to 16.3) |
| Massachusetts | 7914 (5453 to 11225) | 57.9 (40.7 to 80.5) | -14.7% (-32.6 to 4.9) | 187573 (143776 to 238082) | 1589.7 (1216.5 to 2005.1) | -6.1% (-21.7 to 10.2) |
| Michigan | 16494 (11825 to 23045) | 83.6 (60.4 to 116.4) | -5.8% (-26.2 to 18.9) | 391517 (304354 to 491057) | 2239.4 (1748.8 to 2782.1) | 3.5% (-14.1 to 23.0) |
| Minnesota | 5847 (4118 to 8365) | 55.2 (39.3 to 77.8) | -12.0% (-30.9 to 9.5) | 143670 (111157 to 180883) | 1540.3 (1196.2 to 1925.9) | -0.9% (-17.9 to 16.7) |
| Mississippi | 5260 (3787 to 7031) | 102.5 (74.6 to 136.8) | 13.4% (-10.3 to 40.1) | 133521 (103931 to 167184) | 2810.3 (2193.7 to 3507.7) | 23.4% (2.6 to 46.7) |
| Missouri | 9737 (6733 to 13638) | 81.4 (57.0 to 112.1) | 4.6% (-16.2 to 30.6) | 234424 (181833 to 294080) | 2209.7 (1730.0 to 2761.6) | 14.9% (-2.7 to 36.0) |
| Montana | 1389 (980 to 1923) | 64.2 (45.7 to 87.9) | -1.0% (-21.3 to 21.1) | 33294 (25849 to 42121) | 1720.1 (1353.4 to 2153.1) | 7.7% (-8.4 to 25.4) |
| Nebraska | 2379 (1684 to 3283) | 65.2 (46.5 to 89.2) | 1.1% (-19.1 to 25.5) | 56091 (43360 to 71393) | 1786.4 (1393.8 to 2262.1) | 12.1% (-4.8 to 31.4) |
| Nevada | 3658 (2550 to 5137) | 75.8 (52.7 to 106.9) | -13.1% (-30.3 to 7.8) | 94890 (73024 to 121441) | 1953.7 (1517.3 to 2491.7) | 2.3% (-13.3 to 21.6) |
| New Hampshire | 1836 (1275 to 2502) | 67.6 (47.2 to 91.6) | -9.4% (-29.1 to 13.4) | 45525 (35131 to 57892) | 1806.9 (1397.2 to 2288.9) | 0.0% (-16.7 to 19.6) |
| New Jersey | 12398 (8426 to 17355) | 68.8 (47.9 to 95.2) | -18.7% (-34.8 to 0.4) | 291406 (227312 to 366703) | 1872.8 (1474.1 to 2332.0) | -9.6% (-23.1 to 5.8) |
| New Mexico | 3063 (2197 to 4064) | 73.4 (53.3 to 96.6) | -6.7% (-26.0 to 17.0) | 76683 (60956 to 95181) | 2075.5 (1667.6 to 2549.0) | 5.6% (-12.0 to 23.6) |
| New York | 26805 (17644 to 37872) | 66.2 (45.0 to 92.5) | -13.7% (-31.8 to 8.3) | 630300 (485227 to 786672) | 1851.1 (1437.4 to 2305.5) | -1.9% (-17.6 to 15.0) |
| North Carolina | 14996 (10851 to 20453) | 81.6 (59.3 to 111.1) | -8.5% (-27.0 to 13.3) | 385820 (308173 to 481455) | 2245.2 (1811.4 to 2787.9) | 0.8% (-14.8 to 18.8) |
| North Dakota | 996 (702 to 1350) | 67.7 (48.8 to 90.2) | -2.3% (-21.1 to 20.5) | 22895 (17932 to 28655) | 1897.1 (1501.5 to 2353.7) | 11.5% (-6.2 to 31.5) |
| Ohio | 20104 (14286 to 27616) | 87.7 (63.3 to 118.2) | -8.9% (-27.3 to 11.3) | 483128 (379585 to 608126) | 2382.1 (1892.2 to 2987.5) | 2.0% (-14.4 to 19.8) |
| Oklahoma | 6609 (4668 to 8987) | 95.5 (67.6 to 128.8) | 20.9% (-2.8 to 51.0) | 160771 (125842 to 200988) | 2560.7 (2027.9 to 3183.2) | 29.7% (9.0 to 54.0) |
| Oregon | 5058 (3632 to 6954) | 62.7 (45.6 to 85.4) | -8.7% (-28.6 to 13.3) | 123793 (97146 to 155805) | 1711.7 (1348.6 to 2145.8) | 1.6% (-14.9 to 19.4) |
| Pennsylvania | 21077 (14458 to 29036) | 75.0 (52.9 to 103.0) | -25.0% (-40.2 to -6.0) | 482014 (373740 to 609320) | 2011.3 (1579.8 to 2525.8) | -16.0% (-29.9 to -0.2) |
| Rhode Island | 1497 (1011 to 2145) | 66.7 (46.4 to 93.2) | -9.5% (-26.8 to 12.5) | 33908 (25937 to 42707) | 1782.1 (1375.9 to 2234.1) | -0.9% (-17.1 to 15.9) |
| South Carolina | 7849 (5733 to 10665) | 86.5 (63.4 to 117.4) | -8.9% (-26.7 to 12.4) | 204455 (161131 to 255091) | 2413.5 (1906.4 to 3011.0) | 0.9% (-15.5 to 18.6) |
| South Dakota | 1207 (860 to 1674) | 69.1 (50.3 to 94.9) | 6.0% (-13.6 to 29.1) | 27803 (21839 to 35082) | 1894.1 (1509.8 to 2358.8) | 16.9% (-0.6 to 36.7) |
| Tennessee | 11138 (7892 to 15394) | 92.9 (66.2 to 127.3) | 4.8% (-17.0 to 31.4) | 280540 (219544 to 357207) | 2519.6 (1985.1 to 3180.5) | 14.7% (-4.1 to 36.4) |
| Texas | 31157 (21884 to 42482) | 76.6 (54.1 to 104.1) | -9.7% (-28.7 to 11.3) | 831356 (648288 to 1045213) | 2114.6 (1653.6 to 2641.1) | -0.1% (-16.5 to 19.2) |
| Utah | 2721 (1981 to 3748) | 70.3 (51.2 to 96.7) | -7.9% (-25.4 to 11.9) | 69735 (55341 to 86881) | 1848.0 (1475.6 to 2293.8) | -0.4% (-15.5 to 15.8) |
| Vermont | 857 (611 to 1194) | 62.1 (45.0 to 85.8) | -13.8% (-30.1 to 4.8) | 20320 (15640 to 25835) | 1658.3 (1291.0 to 2085.8) | -4.7% (-19.8 to 10.6) |
| Virginia | 10747 (7458 to 14614) | 73.1 (51.1 to 98.8) | -5.9% (-24.8 to 17.0) | 277346 (219040 to 349966) | 2007.1 (1591.6 to 2524.2) | 5.0% (-11.7 to 24.4) |
| Washington | 7802 (5468 to 10831) | 59.8 (42.4 to 82.6) | -10.7% (-29.3 to 11.5) | 198239 (153767 to 250579) | 1645.2 (1282.2 to 2081.9) | -0.4% (-16.2 to 16.6) |
| West Virginia | 4113 (2925 to 5616) | 103.8 (74.7 to 140.2) | 4.6% (-17.2 to 28.4) | 99094 (78119 to 124121) | 2813.5 (2241.0 to 3497.5) | 14.9% (-4.8 to 36.0) |
| Wisconsin | 7525 (5312 to 10494) | 65.4 (47.0 to 91.1) | -20.1% (-38.0 to 0.9) | 178119 (138407 to 225320) | 1739.9 (1362.2 to 2186.0) | -10.3% (-25.9 to 5.9) |
| Wyoming | 651 (460 to 891) | 60.3 (43.0 to 81.8) | -6.9% (-24.5 to 12.9) | 16258 (12793 to 20335) | 1633.7 (1296.1 to 2017.5) | 2.6% (-12.1 to 18.9) |

Data in parentheses are 95% uncertainty intervals. DALYs= disability-adjusted life-years.

**Table S3.** Deaths and DALYs attributable to high LDL cholesterol in 2019 and percentage change of age-standardised rates from 1990 to 2019, by location.

|  | Deaths |  |  | DALYs |  |  |
| --- | --- | --- | --- | --- | --- | --- |
|  | Counts | Age-standardised rates per 100,000 people | Percentage change in age-standardised rates, 1990-2019 | Counts | Age-standardised rates per 100,000 people | Percentage change in age-standardised rates, 1990-2019 |
| United States | 226343 (158853 to 304367) | 38.0 (27.7 to 50.0) | -64.9% (-68.0 to -61.8) | 4167272 (3307627 to 5112983) | 805.0 (656.3 to 968.3) | -62.4% (-65.1 to -59.9) |
| Alabama | 4285 (2978 to 5880) | 50.3 (35.7 to 68.1) | -55.5% (-62.7 to -46.9) | 87308 (66892 to 112387) | 1148.7 (897.1 to 1459.9) | -51.6% (-59.5 to -41.9) |
| Alaska | 324 (244 to 425) | 33.6 (24.3 to 45.0) | -70.2% (-74.5 to -65.4) | 7278 (5777 to 9172) | 711.7 (567.7 to 895.6) | -64.2% (-69.3 to -57.9) |
| Arizona | 4718 (3073 to 6594) | 35.0 (23.8 to 47.6) | -62.4% (-68.9 to -55.1) | 83265 (61178 to 107748) | 720.8 (543.4 to 919.5) | -60.6% (-67.1 to -52.9) |
| Arkansas | 2863 (2034 to 3852) | 53.5 (39.4 to 70.4) | -53.8% (-61.5 to -46.1) | 58016 (44419 to 73773) | 1253.0 (971.1 to 1574.4) | -48.5% (-57.1 to -39.0) |
| California | 21811 (14597 to 30604) | 31.2 (21.7 to 42.5) | -69.9% (-75.1 to -64.8) | 378557 (285902 to 487951) | 621.9 (476.0 to 789.9) | -67.9% (-73.6 to -62.2) |
| Colorado | 2544 (1749 to 3495) | 29.5 (20.3 to 40.4) | -65.5% (-71.5 to -59.2) | 47279 (35532 to 61109) | 581.1 (442.3 to 747.1) | -63.9% (-70.1 to -56.8) |
| Connecticut | 2346 (1507 to 3337) | 29.3 (19.9 to 40.4) | -68.7% (-74.4 to -62.8) | 38634 (28766 to 51025) | 586.9 (446.0 to 758.9) | -67.5% (-73.5 to -61.0) |
| Delaware | 753 (519 to 1020) | 40.4 (28.5 to 53.6) | -64.9% (-70.1 to -59.5) | 13507 (10248 to 17005) | 824.3 (640.6 to 1026.3) | -63.1% (-68.5 to -57.0) |
| District of Columbia | 410 (284 to 569) | 40.4 (28.6 to 55.5) | -60.4% (-67.8 to -52.4) | 7896 (5943 to 10470) | 895.8 (678.3 to 1187.4) | -62.6% (-69.4 to -55.1) |
| Florida | 17691 (11680 to 24973) | 35.9 (25.0 to 48.7) | -62.9% (-69.3 to -56.8) | 297907 (220815 to 389552) | 773.5 (590.5 to 980.3) | -59.9% (-66.8 to -52.8) |
| Georgia | 6275 (4485 to 8508) | 41.3 (29.5 to 56.3) | -64.7% (-70.6 to -58.0) | 133342 (102681 to 171520) | 893.2 (695.3 to 1136.3) | -62.9% (-69.0 to -55.7) |
| Hawaii | 835 (562 to 1177) | 26.1 (18.2 to 35.5) | -66.3% (-72.2 to -60.1) | 14843 (10981 to 19263) | 588.8 (447.1 to 748.2) | -62.1% (-69.0 to -54.1) |
| Idaho | 973 (666 to 1359) | 34.4 (24.0 to 47.3) | -62.1% (-68.5 to -55.4) | 17406 (12949 to 22515) | 674.7 (515.7 to 859.2) | -60.8% (-67.6 to -53.2) |
| Illinois | 8784 (6109 to 12080) | 36.9 (26.6 to 50.0) | -67.6% (-72.9 to -62.1) | 161268 (123908 to 208939) | 784.9 (615.8 to 1011.0) | -65.2% (-70.8 to -58.6) |
| Indiana | 5118 (3469 to 6991) | 44.1 (30.9 to 59.1) | -61.1% (-67.7 to -54.0) | 100096 (76681 to 128758) | 976.6 (757.4 to 1237.3) | -56.4% (-63.9 to -48.2) |
| Iowa | 2515 (1687 to 3532) | 38.5 (27.1 to 52.4) | -60.4% (-67.1 to -53.2) | 42591 (31577 to 55118) | 813.4 (619.6 to 1041.3) | -56.5% (-64.2 to -47.5) |
| Kansas | 2033 (1383 to 2787) | 37.7 (26.6 to 50.4) | -59.6% (-66.4 to -52.3) | 36561 (27428 to 46951) | 799.7 (615.1 to 1015.3) | -56.4% (-63.8 to -47.6) |
| Kentucky | 3811 (2765 to 5234) | 50.0 (37.0 to 67.1) | -58.0% (-64.4 to -50.4) | 79460 (62359 to 102950) | 1151.5 (911.3 to 1465.1) | -53.5% (-60.8 to -44.5) |
| Louisiana | 3590 (2551 to 4841) | 47.9 (34.6 to 64.5) | -61.3% (-67.9 to -54.1) | 75353 (57672 to 95891) | 1115.5 (864.5 to 1408.5) | -57.4% (-64.9 to -49.2) |
| Maine | 1049 (690 to 1459) | 35.5 (24.0 to 48.4) | -65.0% (-71.1 to -59.1) | 18122 (13314 to 23842) | 709.2 (538.9 to 914.4) | -63.9% (-70.1 to -57.1) |
| Maryland | 4209 (2833 to 5771) | 39.4 (27.0 to 53.4) | -62.3% (-69.4 to -55.9) | 78333 (58019 to 101834) | 814.6 (611.0 to 1048.1) | -59.9% (-67.3 to -52.2) |
| Massachusetts | 4191 (2735 to 6005) | 30.4 (20.8 to 42.8) | -68.5% (-74.0 to -62.7) | 70984 (52441 to 94523) | 618.5 (474.6 to 812.9) | -67.7% (-73.4 to -61.4) |
| Michigan | 8646 (5939 to 11890) | 45.2 (31.8 to 60.6) | -62.1% (-68.4 to -55.3) | 158572 (117533 to 202922) | 963.3 (734.3 to 1214.3) | -59.2% (-66.1 to -51.3) |
| Minnesota | 2776 (1886 to 3943) | 26.3 (18.3 to 36.2) | -70.5% (-75.8 to -65.0) | 48203 (35323 to 63010) | 536.7 (400.2 to 693.5) | -68.6% (-74.2 to -62.1) |
| Mississippi | 2609 (1862 to 3479) | 53.2 (38.6 to 70.0) | -58.9% (-65.6 to -50.9) | 55272 (42173 to 71002) | 1252.0 (979.4 to 1596.7) | -55.0% (-62.4 to -45.7) |
| Missouri | 5226 (3656 to 7065) | 45.4 (33.0 to 60.3) | -59.0% (-65.7 to -51.5) | 100306 (76857 to 127960) | 1014.9 (794.0 to 1281.9) | -54.0% (-61.5 to -44.6) |
| Montana | 712 (483 to 1003) | 34.3 (24.0 to 47.2) | -61.2% (-67.5 to -53.7) | 12876 (9585 to 17008) | 714.3 (543.0 to 919.7) | -57.4% (-64.8 to -49.0) |
| Nebraska | 1151 (766 to 1640) | 31.6 (21.8 to 42.9) | -66.9% (-72.6 to -60.9) | 19519 (14695 to 25581) | 644.2 (496.9 to 826.0) | -65.0% (-70.9 to -58.3) |
| Nevada | 2026 (1419 to 2769) | 43.8 (30.6 to 59.6) | -67.2% (-72.4 to -61.6) | 40205 (30459 to 52778) | 865.1 (660.3 to 1129.2) | -63.0% (-69.2 to -55.6) |
| New Hampshire | 925 (610 to 1312) | 34.6 (23.3 to 48.5) | -66.1% (-72.0 to -59.5) | 16269 (12142 to 21468) | 673.0 (515.2 to 872.7) | -66.1% (-71.9 to -59.0) |
| New Jersey | 6471 (4305 to 9145) | 35.2 (24.1 to 48.7) | -67.6% (-73.1 to -61.7) | 107797 (80765 to 141626) | 701.3 (535.1 to 911.4) | -66.5% (-71.9 to -60.3) |
| New Mexico | 1424 (952 to 1981) | 34.9 (24.1 to 48.0) | -60.4% (-66.7 to -53.3) | 25631 (19356 to 33920) | 724.6 (560.2 to 931.3) | -56.7% (-63.9 to -48.4) |
| New York | 15538 (10047 to 21999) | 38.0 (25.9 to 51.7) | -68.6% (-74.1 to -62.8) | 257151 (190230 to 333747) | 769.7 (586.5 to 980.7) | -67.6% (-73.1 to -61.7) |
| North Carolina | 7158 (4942 to 9744) | 40.4 (28.6 to 54.6) | -65.2% (-71.1 to -58.8) | 141811 (108142 to 180935) | 873.2 (675.4 to 1098.1) | -63.4% (-69.4 to -56.3) |
| North Dakota | 505 (336 to 707) | 34.6 (24.8 to 46.1) | -63.5% (-69.0 to -58.0) | 8682 (6649 to 11299) | 756.8 (596.4 to 962.7) | -58.9% (-65.4 to -51.8) |
| Ohio | 9790 (6820 to 13384) | 43.8 (31.2 to 58.8) | -62.3% (-68.2 to -55.7) | 180447 (137506 to 233834) | 943.1 (732.3 to 1212.6) | -59.6% (-66.3 to -52.2) |
| Oklahoma | 3529 (2464 to 4779) | 53.1 (38.0 to 70.8) | -54.8% (-62.5 to -46.8) | 70203 (53678 to 89329) | 1196.2 (924.1 to 1495.4) | -50.4% (-59.5 to -41.0) |
| Oregon | 2314 (1537 to 3246) | 29.4 (20.1 to 40.4) | -69.7% (-75.0 to -63.9) | 40171 (29638 to 52530) | 584.2 (443.1 to 746.6) | -68.2% (-73.6 to -62.2) |
| Pennsylvania | 10859 (7212 to 15509) | 38.8 (26.8 to 53.7) | -65.4% (-71.4 to -59.2) | 185501 (136125 to 243077) | 811.2 (609.7 to 1028.6) | -63.5% (-69.7 to -56.5) |
| Rhode Island | 849 (560 to 1201) | 37.1 (25.5 to 51.0) | -65.7% (-71.5 to -59.7) | 13545 (10020 to 17683) | 721.6 (556.4 to 926.7) | -66.1% (-71.6 to -59.7) |
| South Carolina | 3762 (2613 to 5059) | 44.1 (31.3 to 58.5) | -63.2% (-69.3 to -56.3) | 78440 (59984 to 99717) | 1015.3 (788.9 to 1273.3) | -60.1% (-66.9 to -52.0) |
| South Dakota | 673 (460 to 939) | 38.9 (28.0 to 52.6) | -60.5% (-66.6 to -54.1) | 11605 (8842 to 15032) | 829.8 (646.7 to 1045.2) | -56.8% (-63.4 to -48.7) |
| Tennessee | 5857 (4071 to 8039) | 51.2 (36.4 to 69.7) | -58.6% (-65.6 to -50.7) | 119815 (91105 to 155093) | 1154.1 (893.9 to 1478.1) | -55.0% (-62.7 to -45.7) |
| Texas | 15599 (10945 to 21217) | 38.9 (27.6 to 52.6) | -63.8% (-69.7 to -57.6) | 318890 (243019 to 409116) | 833.8 (641.8 to 1063.0) | -61.4% (-67.7 to -54.5) |
| Utah | 1213 (823 to 1699) | 31.8 (21.6 to 44.4) | -63.5% (-69.8 to -57.2) | 21245 (16020 to 27805) | 574.5 (435.7 to 748.1) | -64.1% (-70.0 to -57.4) |
| Vermont | 465 (320 to 640) | 34.1 (24.2 to 45.8) | -66.5% (-71.3 to -61.2) | 7995 (6140 to 10275) | 679.4 (532.2 to 851.8) | -65.2% (-70.1 to -59.3) |
| Virginia | 5078 (3541 to 6915) | 35.4 (25.1 to 47.5) | -67.2% (-72.8 to -61.3) | 99015 (75764 to 126677) | 749.3 (580.8 to 952.9) | -64.9% (-70.9 to -58.3) |
| Washington | 3805 (2575 to 5240) | 29.6 (20.4 to 40.4) | -68.7% (-74.2 to -63.0) | 67773 (50029 to 88384) | 583.0 (436.7 to 746.3) | -67.4% (-73.0 to -61.3) |
| West Virginia | 1929 (1328 to 2635) | 50.9 (36.4 to 68.1) | -58.0% (-65.0 to -50.8) | 36200 (27059 to 46788) | 1113.7 (867.3 to 1405.5) | -56.0% (-63.3 to -47.7) |
| Wisconsin | 3966 (2599 to 5591) | 34.8 (23.6 to 47.7) | -66.3% (-72.0 to -60.3) | 69372 (52119 to 90050) | 704.9 (536.7 to 895.7) | -63.9% (-70.2 to -57.1) |
| Wyoming | 358 (253 to 491) | 34.0 (24.8 to 46.1) | -63.4% (-68.1 to -58.1) | 6752 (5256 to 8748) | 714.2 (568.1 to 908.6) | -60.1% (-65.4 to -53.9) |

Data in parentheses are 95% uncertainty intervals. DALYs= disability-adjusted life-years.

**Table S4**. Deaths and DALYs attributable to high systolic blood pressure in 2019 and percentage change of age-standardised rates from 1990 to 2019, by location.

|  | Deaths |  |  | DALYs |  |  |
| --- | --- | --- | --- | --- | --- | --- |
|  | Counts | Age-standardised rates per 100,000 people | Percentage change in age-standardised rates, 1990-2019 | Counts | Age-standardised rates per 100,000 people | Percentage change in age-standardised rates, 1990-2019 |
| United States | 495201 (407465 to 574654) | 81.6 (68.6 to 94.2) | -45.9% (-49.6 to -42.1) | 9102668 (7925411 to 10254601) | 1684.1 (1478.7 to 1880.9) | -42.7% (-45.9 to -39.4) |
| Alabama | 9737 (7613 to 11843) | 109.9 (86.6 to 133.8) | -34.2% (-45.3 to -21.8) | 189353 (153664 to 226334) | 2340.7 (1889.2 to 2796.1) | -30.8% (-41.9 to -17.6) |
| Alaska | 710 (584 to 856) | 74.6 (60.7 to 90.4) | -55.5% (-62.6 to -47.3) | 15268 (12586 to 18101) | 1499.1 (1242.9 to 1781.5) | -47.9% (-55.9 to -38.4) |
| Arizona | 10114 (7794 to 12538) | 73.2 (56.6 to 90.4) | -43.0% (-53.0 to -31.1) | 180880 (145680 to 218621) | 1485.4 (1204.7 to 1791.5) | -39.6% (-49.6 to -28.3) |
| Arkansas | 6038 (4751 to 7308) | 108.2 (86.0 to 130.6) | -33.4% (-44.5 to -20.7) | 116447 (94746 to 139161) | 2350.2 (1914.4 to 2816.5) | -28.0% (-39.6 to -14.2) |
| California | 48678 (37990 to 59780) | 69.0 (54.3 to 83.9) | -51.3% (-59.2 to -42.9) | 860874 (710577 to 1022248) | 1383.0 (1143.3 to 1647.8) | -47.9% (-55.7 to -39.1) |
| Colorado | 5816 (4426 to 7188) | 66.5 (50.9 to 81.9) | -45.6% (-55.8 to -34.4) | 107353 (86242 to 129247) | 1276.8 (1025.7 to 1535.0) | -43.3% (-53.3 to -32.7) |
| Connecticut | 5072 (3857 to 6352) | 62.5 (49.0 to 78.1) | -52.6% (-61.4 to -43.0) | 85322 (68638 to 103056) | 1246.5 (1005.9 to 1506.4) | -50.1% (-58.6 to -40.3) |
| Delaware | 1594 (1260 to 1946) | 83.5 (66.5 to 101.5) | -49.8% (-57.5 to -41.0) | 29497 (24172 to 34870) | 1710.3 (1399.5 to 2026.5) | -45.6% (-53.7 to -36.6) |
| District of Columbia | 1021 (803 to 1242) | 100.7 (80.3 to 122.6) | -41.7% (-51.5 to -31.1) | 20043 (16461 to 24067) | 2241.7 (1841.8 to 2701.3) | -43.9% (-53.6 to -33.0) |
| Florida | 37463 (28789 to 47585) | 74.4 (58.9 to 92.2) | -44.1% (-54.2 to -33.3) | 651634 (526249 to 792840) | 1595.7 (1282.3 to 1929.3) | -39.5% (-50.0 to -28.4) |
| Georgia | 15341 (12294 to 18541) | 99.4 (79.8 to 120.6) | -41.9% (-51.4 to -31.1) | 318560 (260775 to 381640) | 2073.9 (1697.0 to 2479.6) | -39.7% (-49.5 to -28.7) |
| Hawaii | 1964 (1486 to 2427) | 58.9 (45.9 to 72.4) | -50.9% (-59.3 to -41.0) | 34599 (27515 to 41774) | 1285.0 (1023.8 to 1555.0) | -46.8% (-55.7 to -36.3) |
| Idaho | 2160 (1722 to 2650) | 74.2 (59.3 to 90.8) | -42.5% (-52.2 to -31.2) | 37821 (30998 to 45580) | 1386.3 (1135.7 to 1673.7) | -41.9% (-51.2 to -30.8) |
| Illinois | 20285 (16145 to 25039) | 83.6 (67.0 to 102.8) | -48.1% (-56.8 to -38.4) | 366174 (302673 to 439891) | 1716.3 (1420.0 to 2069.6) | -45.5% (-54.2 to -35.3) |
| Indiana | 11174 (8835 to 13592) | 93.3 (74.0 to 112.8) | -40.5% (-50.5 to -28.9) | 208550 (171146 to 250978) | 1924.3 (1572.6 to 2316.6) | -36.2% (-46.7 to -24.5) |
| Iowa | 5193 (3999 to 6470) | 76.1 (59.6 to 94.5) | -43.8% (-53.5 to -31.8) | 85087 (69033 to 104777) | 1510.4 (1235.1 to 1859.4) | -40.0% (-49.9 to -27.1) |
| Kansas | 4370 (3368 to 5474) | 78.6 (61.8 to 98.0) | -39.8% (-50.1 to -27.9) | 76431 (61940 to 92765) | 1578.9 (1291.6 to 1921.9) | -36.7% (-47.5 to -24.7) |
| Kentucky | 8290 (6689 to 10046) | 105.1 (84.7 to 126.6) | -36.7% (-46.9 to -25.2) | 164198 (134663 to 195280) | 2242.5 (1835.6 to 2665.4) | -32.0% (-43.0 to -20.2) |
| Louisiana | 8702 (6924 to 10528) | 112.9 (90.3 to 136.2) | -37.8% (-48.3 to -25.9) | 174407 (141868 to 209549) | 2469.2 (2008.1 to 2970.9) | -33.9% (-45.1 to -21.3) |
| Maine | 2307 (1780 to 2881) | 75.7 (59.2 to 93.8) | -46.7% (-55.4 to -36.9) | 39754 (31654 to 48273) | 1452.1 (1169.3 to 1760.3) | -45.4% (-54.2 to -35.4) |
| Maryland | 9306 (7273 to 11452) | 86.9 (68.2 to 106.5) | -43.6% (-53.6 to -32.1) | 179016 (144288 to 216069) | 1823.4 (1470.9 to 2194.4) | -39.4% (-49.5 to -28.2) |
| Massachusetts | 9181 (7063 to 11636) | 65.4 (51.1 to 82.5) | -51.3% (-60.2 to -41.5) | 154855 (124757 to 189014) | 1285.9 (1045.9 to 1566.7) | -50.1% (-58.0 to -40.7) |
| Michigan | 17771 (13967 to 21882) | 91.5 (72.6 to 112.1) | -43.7% (-53.7 to -32.3) | 330421 (269126 to 397683) | 1918.7 (1562.8 to 2301.1) | -39.9% (-49.7 to -29.2) |
| Minnesota | 6343 (4908 to 7963) | 58.8 (46.1 to 73.7) | -53.3% (-61.6 to -43.0) | 107795 (87281 to 131178) | 1134.9 (919.4 to 1378.1) | -51.3% (-59.7 to -41.8) |
| Mississippi | 6306 (5127 to 7630) | 124.4 (101.7 to 150.2) | -33.8% (-45.1 to -21.1) | 126799 (105402 to 152916) | 2721.0 (2253.5 to 3282.7) | -29.2% (-40.7 to -15.3) |
| Missouri | 10888 (8552 to 13446) | 91.5 (71.8 to 112.3) | -40.1% (-50.2 to -28.0) | 202680 (164808 to 244964) | 1926.2 (1565.9 to 2320.8) | -35.5% (-45.9 to -22.6) |
| Montana | 1539 (1180 to 1899) | 71.4 (55.2 to 87.4) | -44.6% (-54.7 to -33.6) | 27416 (21950 to 33321) | 1419.3 (1135.4 to 1712.0) | -40.9% (-50.5 to -29.5) |
| Nebraska | 2621 (2010 to 3239) | 70.1 (54.9 to 86.1) | -47.4% (-56.7 to -37.5) | 44058 (35716 to 53008) | 1376.8 (1125.9 to 1653.4) | -45.2% (-54.5 to -35.6) |
| Nevada | 4576 (3696 to 5551) | 97.2 (78.1 to 117.8) | -51.7% (-59.4 to -42.6) | 90883 (75010 to 108188) | 1906.4 (1569.0 to 2259.9) | -44.9% (-53.6 to -34.5) |
| New Hampshire | 1975 (1535 to 2478) | 72.7 (56.6 to 91.1) | -48.7% (-58.1 to -38.5) | 34632 (28092 to 42266) | 1372.7 (1121.8 to 1670.8) | -48.6% (-57.1 to -38.5) |
| New Jersey | 13607 (10463 to 17001) | 74.1 (58.2 to 91.9) | -51.2% (-59.1 to -40.9) | 234796 (191151 to 283156) | 1485.9 (1218.6 to 1807.3) | -49.0% (-57.0 to -39.0) |
| New Mexico | 3026 (2382 to 3754) | 72.2 (57.0 to 88.8) | -41.9% (-51.9 to -31.8) | 55125 (44675 to 66305) | 1483.3 (1201.2 to 1787.7) | -36.8% (-46.8 to -25.0) |
| New York | 30558 (22603 to 38340) | 74.8 (56.5 to 93.0) | -53.6% (-62.1 to -44.2) | 530377 (428486 to 639707) | 1539.2 (1246.2 to 1855.6) | -51.0% (-59.2 to -42.0) |
| North Carolina | 15917 (12730 to 19664) | 87.4 (70.0 to 107.8) | -47.0% (-55.7 to -36.8) | 308748 (251856 to 374603) | 1809.3 (1477.2 to 2201.1) | -44.9% (-53.6 to -34.6) |
| North Dakota | 1055 (808 to 1288) | 70.4 (55.8 to 84.8) | -46.7% (-55.1 to -37.6) | 17978 (14705 to 21401) | 1471.0 (1215.7 to 1745.3) | -40.9% (-49.6 to -30.7) |
| Ohio | 21700 (17243 to 26323) | 94.5 (75.5 to 113.4) | -41.5% (-50.9 to -30.8) | 395253 (325260 to 465312) | 1953.0 (1611.5 to 2307.2) | -38.1% (-47.1 to -27.6) |
| Oklahoma | 7580 (5994 to 9209) | 110.8 (88.0 to 134.3) | -31.4% (-43.0 to -18.2) | 146951 (120838 to 174930) | 2369.1 (1936.5 to 2812.5) | -26.0% (-38.1 to -12.2) |
| Oregon | 5519 (4327 to 6816) | 67.6 (53.5 to 84.0) | -51.2% (-59.8 to -41.5) | 94586 (77013 to 114280) | 1287.2 (1054.1 to 1565.9) | -49.8% (-58.2 to -41.0) |
| Pennsylvania | 23194 (17565 to 28819) | 81.5 (64.1 to 100.0) | -47.7% (-56.5 to -37.2) | 401372 (324214 to 488141) | 1662.5 (1344.3 to 2028.8) | -45.0% (-54.0 to -34.5) |
| Rhode Island | 1654 (1256 to 2070) | 72.1 (56.2 to 89.6) | -50.0% (-58.6 to -40.5) | 27308 (22122 to 33004) | 1405.3 (1147.1 to 1699.7) | -49.0% (-57.1 to -39.7) |
| South Carolina | 8659 (6821 to 10462) | 97.2 (76.7 to 117.4) | -43.6% (-53.9 to -32.6) | 172571 (139652 to 204991) | 2079.1 (1695.0 to 2462.3) | -41.3% (-50.9 to -30.4) |
| South Dakota | 1354 (1047 to 1654) | 75.6 (59.7 to 91.6) | -42.7% (-51.7 to -31.9) | 22969 (18646 to 27504) | 1537.9 (1250.2 to 1832.8) | -38.7% (-48.0 to -27.4) |
| Tennessee | 12428 (9719 to 15333) | 105.1 (82.8 to 129.8) | -38.5% (-48.6 to -25.9) | 245238 (200936 to 299167) | 2234.8 (1839.3 to 2729.3) | -34.8% (-45.5 to -22.1) |
| Texas | 34868 (26821 to 42552) | 86.3 (66.5 to 105.2) | -42.9% (-52.7 to -32.2) | 707737 (568396 to 842643) | 1815.1 (1458.3 to 2158.4) | -39.3% (-49.0 to -29.2) |
| Utah | 2825 (2260 to 3445) | 73.4 (58.5 to 89.5) | -42.0% (-51.9 to -31.3) | 50245 (40833 to 60441) | 1332.0 (1079.8 to 1603.0) | -42.4% (-51.7 to -31.5) |
| Vermont | 982 (765 to 1198) | 70.4 (56.2 to 85.1) | -50.0% (-57.8 to -41.3) | 16828 (13791 to 19928) | 1357.8 (1117.1 to 1608.4) | -48.4% (-55.9 to -40.1) |
| Virginia | 11449 (8848 to 14033) | 78.3 (61.0 to 96.1) | -49.4% (-58.6 to -40.0) | 217813 (177555 to 261672) | 1584.3 (1291.8 to 1898.5) | -46.9% (-55.7 to -37.4) |
| Washington | 8675 (6716 to 10784) | 66.2 (51.7 to 82.1) | -50.5% (-58.7 to -41.0) | 154596 (124247 to 188339) | 1276.4 (1031.8 to 1558.4) | -48.7% (-56.6 to -39.5) |
| West Virginia | 4020 (3134 to 5006) | 101.5 (79.7 to 125.9) | -38.6% (-48.9 to -26.9) | 73798 (60409 to 89467) | 2099.0 (1725.2 to 2550.2) | -36.5% (-46.6 to -24.5) |
| Wisconsin | 8840 (6835 to 10873) | 76.2 (59.5 to 92.9) | -47.0% (-56.4 to -36.6) | 153465 (124937 to 184514) | 1488.1 (1213.0 to 1788.2) | -44.4% (-53.7 to -33.4) |
| Wyoming | 756 (597 to 932) | 70.2 (55.8 to 86.1) | -46.8% (-54.3 to -37.5) | 14109 (11613 to 16692) | 1424.6 (1181.2 to 1676.8) | -42.4% (-49.9 to -33.0) |

Data in parentheses are 95% uncertainty intervals. DALYs= disability-adjusted life-years.

**Table S5.** Deaths and DALYs attributable to high body mass index in 2019 and percentage change of age-standardised rates from 1990 to 2019, by location.

|  | Deaths |  |  | DALYs |  |  |
| --- | --- | --- | --- | --- | --- | --- |
|  | Counts | Age-standardised rates per 100,000 people | Percentage change in age-standardised rates, 1990-2019 | Counts | Age-standardised rates per 100,000 people | Percentage change in age-standardised rates, 1990-2019 |
| United States | 393859 (257610 to 528439) | 68.5 (45.7 to 90.5) | -5.8% (-15.3 to 10.0) | 12536102 (8694735 to 16165298) | 2498.2 (1759.9 to 3203.1) | 6.5% (-3.7 to 24.7) |
| Alabama | 7601 (4975 to 10440) | 89.7 (59.6 to 122.4) | 9.0% (-10.5 to 36.2) | 246273 (171801 to 319616) | 3259.8 (2299.6 to 4217.7) | 19.8% (2.9 to 44.0) |
| Alaska | 668 (438 to 892) | 65.4 (42.3 to 88.5) | -17.5% (-30.7 to 2.5) | 24551 (17169 to 31925) | 2342.0 (1646.9 to 3057.6) | 0.4% (-12.2 to 19.0) |
| Arizona | 7694 (4745 to 10850) | 59.5 (36.9 to 83.1) | 3.7% (-16.0 to 34.3) | 249704 (167937 to 333580) | 2264.6 (1562.2 to 3016.0) | 16.2% (-1.0 to 42.3) |
| Arkansas | 4697 (3039 to 6389) | 89.6 (59.2 to 120.5) | 17.8% (-4.6 to 53.5) | 145501 (100837 to 189205) | 3177.3 (2236.6 to 4099.9) | 25.6% (6.4 to 54.4) |
| California | 37108 (23183 to 51472) | 56.1 (35.4 to 77.0) | -8.6% (-24.6 to 18.2) | 1207219 (827835 to 1592483) | 2083.5 (1442.3 to 2722.5) | 5.7% (-10.0 to 29.5) |
| Colorado | 4538 (2824 to 6305) | 52.2 (32.7 to 72.3) | -4.7% (-21.9 to 23.2) | 151167 (101111 to 202165) | 1882.4 (1272.6 to 2523.7) | 6.6% (-8.7 to 29.5) |
| Connecticut | 4119 (2509 to 5817) | 54.8 (34.4 to 77.0) | -14.5% (-29.8 to 10.3) | 126473 (84644 to 168693) | 2054.9 (1409.0 to 2717.8) | 0.4% (-13.3 to 21.4) |
| Delaware | 1285 (833 to 1752) | 70.3 (46.1 to 94.8) | -16.2% (-28.9 to 2.9) | 41246 (28784 to 53706) | 2595.6 (1842.1 to 3344.8) | 0.0% (-12.4 to 17.6) |
| District of Columbia | 820 (531 to 1126) | 85.7 (56.4 to 116.6) | -17.7% (-31.8 to 0.8) | 24751 (17075 to 32676) | 2881.0 (2016.9 to 3801.2) | -16.8% (-28.1 to -1.3) |
| Florida | 27826 (16917 to 38594) | 62.0 (39.1 to 84.9) | 0.6% (-15.9 to 29.6) | 863836 (576569 to 1142538) | 2403.0 (1630.3 to 3138.2) | 14.2% (-1.2 to 38.5) |
| Georgia | 12484 (8043 to 17129) | 79.8 (51.8 to 109.5) | -3.4% (-20.8 to 22.3) | 428927 (301910 to 564343) | 2850.2 (2034.1 to 3745.4) | 7.9% (-8.0 to 32.0) |
| Hawaii | 1495 (914 to 2164) | 50.1 (31.4 to 70.9) | 0.0% (-18.4 to 28.4) | 48416 (32834 to 65878) | 2019.7 (1393.7 to 2722.5) | 13.9% (-3.3 to 39.9) |
| Idaho | 1691 (1053 to 2371) | 59.7 (37.6 to 83.2) | 1.5% (-16.8 to 28.6) | 53805 (36375 to 71595) | 2119.5 (1456.7 to 2796.7) | 11.3% (-4.2 to 34.2) |
| Illinois | 15722 (10142 to 21345) | 69.2 (45.1 to 93.0) | -13.4% (-29.2 to 9.2) | 491306 (339679 to 642453) | 2479.2 (1733.0 to 3228.4) | -1.2% (-16.1 to 19.2) |
| Indiana | 9108 (5758 to 12699) | 79.6 (51.1 to 109.0) | 2.7% (-16.2 to 29.3) | 288860 (204085 to 381319) | 2849.5 (2037.9 to 3731.9) | 15.0% (-1.5 to 37.4) |
| Iowa | 4062 (2572 to 5716) | 65.9 (42.3 to 91.4) | 0.5% (-18.3 to 27.4) | 116905 (80525 to 154261) | 2344.8 (1648.3 to 3079.8) | 12.3% (-4.0 to 34.9) |
| Kansas | 3461 (2177 to 4882) | 67.0 (43.2 to 93.6) | 8.2% (-12.5 to 39.0) | 107936 (74309 to 142829) | 2444.5 (1707.7 to 3227.4) | 18.2% (0.9 to 46.1) |
| Kentucky | 6720 (4331 to 9140) | 87.8 (57.4 to 119.2) | 12.7% (-7.6 to 43.4) | 217795 (150562 to 285349) | 3158.3 (2205.6 to 4118.4) | 23.3% (5.5 to 48.9) |
| Louisiana | 7151 (4658 to 9743) | 96.1 (63.3 to 130.6) | -1.1% (-18.5 to 24.3) | 228843 (159075 to 297988) | 3392.9 (2393.1 to 4400.1) | 9.6% (-6.0 to 32.2) |
| Maine | 1913 (1204 to 2687) | 65.7 (42.1 to 91.6) | -4.1% (-20.7 to 20.3) | 58057 (39212 to 76586) | 2343.4 (1595.1 to 3079.4) | 8.2% (-6.8 to 29.4) |
| Maryland | 7745 (5080 to 10696) | 74.6 (49.6 to 102.7) | -5.9% (-22.9 to 18.5) | 248448 (173574 to 324164) | 2660.9 (1866.7 to 3455.2) | 6.4% (-7.6 to 28.4) |
| Massachusetts | 7351 (4450 to 10569) | 56.2 (35.2 to 80.6) | -14.8% (-30.6 to 6.4) | 221466 (148360 to 299070) | 2026.1 (1384.6 to 2717.1) | -3.6% (-16.7 to 16.0) |
| Michigan | 14608 (9376 to 20027) | 78.7 (51.8 to 106.9) | -3.2% (-21.0 to 20.6) | 447367 (307383 to 584241) | 2799.9 (1960.6 to 3628.1) | 8.1% (-7.5 to 29.2) |
| Minnesota | 5378 (3282 to 7569) | 52.9 (33.0 to 74.2) | -13.7% (-29.6 to 9.9) | 167881 (114573 to 221401) | 1930.2 (1332.4 to 2532.0) | 0.0% (-14.4 to 22.8) |
| Mississippi | 5033 (3360 to 6819) | 102.9 (69.6 to 138.6) | 14.1% (-6.8 to 43.4) | 156943 (112157 to 204801) | 3546.9 (2553.5 to 4603.4) | 21.2% (2.9 to 46.8) |
| Missouri | 8569 (5402 to 11886) | 76.5 (49.0 to 105.0) | 4.3% (-14.8 to 31.6) | 268032 (181196 to 352873) | 2764.0 (1896.8 to 3620.8) | 16.9% (-0.7 to 42.2) |
| Montana | 1260 (794 to 1740) | 61.4 (39.2 to 84.2) | 4.2% (-14.4 to 32.2) | 38432 (25789 to 50686) | 2186.0 (1488.9 to 2861.2) | 14.9% (-1.9 to 41.1) |
| Nebraska | 2099 (1349 to 2934) | 61.0 (39.8 to 84.4) | -7.0% (-24.0 to 18.4) | 64624 (43880 to 85832) | 2242.0 (1536.1 to 2959.9) | 5.9% (-8.9 to 29.3) |
| Nevada | 3337 (2114 to 4673) | 69.5 (44.0 to 97.4) | -13.0% (-29.7 to 13.1) | 112537 (75465 to 151071) | 2428.7 (1639.3 to 3244.3) | 2.0% (-12.8 to 23.0) |
| New Hampshire | 1690 (1056 to 2326) | 63.7 (40.1 to 87.0) | -11.7% (-27.4 to 10.5) | 53261 (36481 to 70613) | 2267.8 (1580.9 to 2996.5) | 1.7% (-13.0 to 21.3) |
| New Jersey | 10775 (6673 to 15179) | 63.0 (39.9 to 87.7) | -18.1% (-33.4 to 3.0) | 330422 (227506 to 439721) | 2289.3 (1590.4 to 3038.8) | -5.1% (-17.7 to 13.8) |
| New Mexico | 2465 (1533 to 3510) | 62.6 (39.7 to 88.0) | 9.3% (-10.2 to 40.3) | 80644 (53973 to 108026) | 2388.3 (1634.3 to 3162.9) | 22.1% (4.7 to 49.4) |
| New York | 23526 (15055 to 32482) | 62.4 (41.2 to 84.8) | -20.1% (-35.0 to -0.3) | 733259 (511225 to 967657) | 2352.6 (1654.9 to 3086.8) | -4.8% (-18.5 to 15.1) |
| North Carolina | 13025 (8330 to 17817) | 73.5 (47.7 to 100.0) | -7.4% (-24.0 to 16.0) | 431785 (303221 to 564987) | 2682.1 (1909.8 to 3496.7) | 3.7% (-11.9 to 25.1) |
| North Dakota | 925 (589 to 1286) | 67.7 (43.6 to 91.8) | 1.2% (-15.1 to 26.8) | 26741 (18693 to 35057) | 2435.8 (1720.1 to 3176.6) | 14.4% (-0.9 to 38.4) |
| Ohio | 17433 (11322 to 24044) | 80.7 (53.2 to 110.4) | 0.3% (-16.8 to 26.4) | 537952 (379656 to 708327) | 2886.5 (2070.2 to 3778.8) | 12.1% (-3.5 to 34.6) |
| Oklahoma | 6028 (3834 to 8288) | 92.2 (59.8 to 125.1) | 25.7% (2.5 to 59.6) | 186921 (128862 to 245579) | 3223.6 (2241.3 to 4220.2) | 33.0% (13.0 to 62.2) |
| Oregon | 4471 (2807 to 6295) | 57.8 (36.9 to 80.5) | -8.0% (-24.2 to 17.0) | 139670 (94288 to 185163) | 2096.0 (1438.5 to 2762.0) | 3.4% (-11.0 to 23.9) |
| Pennsylvania | 18913 (11997 to 26191) | 71.8 (46.7 to 98.4) | -10.1% (-25.0 to 11.9) | 558350 (383543 to 731809) | 2567.9 (1817.1 to 3327.0) | 0.2% (-12.9 to 19.2) |
| Rhode Island | 1312 (801 to 1852) | 61.6 (39.0 to 84.9) | -11.3% (-26.5 to 12.2) | 38325 (25684 to 50890) | 2192.7 (1500.1 to 2912.5) | 0.8% (-13.4 to 23.2) |
| South Carolina | 7005 (4579 to 9647) | 80.7 (53.8 to 110.1) | -7.1% (-23.4 to 17.2) | 231830 (162699 to 302464) | 2967.1 (2106.3 to 3844.5) | 4.8% (-10.7 to 26.8) |
| South Dakota | 1088 (686 to 1500) | 67.1 (43.1 to 90.9) | 5.6% (-11.8 to 32.8) | 32082 (21907 to 42471) | 2407.3 (1678.9 to 3178.2) | 17.3% (1.4 to 41.2) |
| Tennessee | 9874 (6310 to 13526) | 86.2 (55.9 to 117.9) | 8.5% (-12.0 to 36.7) | 318201 (218821 to 414169) | 3084.5 (2154.5 to 3998.2) | 18.8% (1.2 to 42.9) |
| Texas | 28931 (18355 to 39767) | 72.1 (46.0 to 99.1) | -1.8% (-19.9 to 24.3) | 998351 (690206 to 1301955) | 2632.4 (1835.6 to 3425.2) | 9.3% (-6.7 to 32.0) |
| Utah | 2234 (1420 to 3132) | 58.3 (37.3 to 81.6) | 2.8% (-15.6 to 32.5) | 76959 (52862 to 103687) | 2094.3 (1439.1 to 2811.2) | 11.6% (-4.0 to 35.4) |
| Vermont | 820 (526 to 1115) | 61.6 (40.5 to 82.8) | -11.2% (-24.2 to 9.0) | 24184 (16787 to 31914) | 2147.4 (1506.0 to 2807.4) | -1.0% (-13.3 to 16.7) |
| Virginia | 9555 (6212 to 13099) | 66.7 (43.8 to 91.4) | -11.1% (-26.3 to 11.7) | 319343 (219229 to 416743) | 2444.7 (1703.4 to 3177.2) | 3.4% (-10.0 to 24.3) |
| Washington | 7149 (4458 to 9932) | 56.2 (35.8 to 77.8) | -7.7% (-25.2 to 18.7) | 233706 (159634 to 310624) | 2062.1 (1418.3 to 2730.1) | 4.8% (-11.2 to 29.3) |
| West Virginia | 3344 (2158 to 4612) | 89.7 (58.8 to 122.3) | 11.1% (-9.0 to 39.5) | 102805 (71222 to 135657) | 3210.9 (2260.8 to 4204.3) | 21.0% (3.1 to 45.3) |
| Wisconsin | 7129 (4461 to 9845) | 64.7 (40.9 to 88.5) | -10.4% (-27.0 to 13.1) | 213819 (145964 to 280768) | 2255.1 (1555.9 to 2942.0) | -0.5% (-14.5 to 19.9) |
| Wyoming | 624 (399 to 861) | 59.7 (38.6 to 82.0) | -5.1% (-18.5 to 16.8) | 20191 (13677 to 26492) | 2184.3 (1494.1 to 2854.3) | 7.0% (-5.8 to 26.7) |

Data in parentheses are 95% uncertainty intervals. DALYs= disability-adjusted life-years.

**Table S6.** Deaths and DALYs attributable to low bone mineral density in 2019 and percentage change of age-standardised rates from 1990 to 2019, by location.

|  | Deaths |  |  | DALYs |  |  |
| --- | --- | --- | --- | --- | --- | --- |
|  | Counts | Age-standardised rates per 100,000 people | Percentage change in age-standardised rates, 1990-2019 | Counts | Age-standardised rates per 100,000 people | Percentage change in age-standardised rates, 1990-2019 |
| United States | 27681 (23019 to 30871) | 4.5 (3.8 to 4.9) | 35.0% (29.8 to 39.0) | 1172666 (926129 to 1476535) | 210.3 (166.3 to 263.8) | 8.7% (6.4 to 11.2) |
| Alabama | 424 (351 to 495) | 4.9 (4.0 to 5.7) | 16.2% (0.7 to 34.5) | 18877 (15096 to 23413) | 228.6 (182.8 to 282.8) | 2.6% (-4.2 to 10.6) |
| Alaska | 49 (41 to 57) | 5.4 (4.4 to 6.2) | -7.6% (-19.1 to 4.5) | 2592 (2047 to 3255) | 260.0 (206.0 to 327.2) | -4.9% (-10.5 to 0.9) |
| Arizona | 796 (635 to 945) | 5.6 (4.5 to 6.7) | 44.6% (23.6 to 68.2) | 29571 (23507 to 36858) | 235.0 (186.4 to 292.7) | 10.4% (3.4 to 18.6) |
| Arkansas | 267 (218 to 316) | 4.8 (3.9 to 5.7) | 29.8% (11.5 to 50.0) | 12013 (9441 to 15140) | 234.4 (184.8 to 294.2) | 8.4% (1.3 to 16.5) |
| California | 2011 (1632 to 2397) | 2.9 (2.4 to 3.4) | 6.2% (-9.1 to 22.6) | 110954 (86305 to 141329) | 176.8 (138.1 to 225.3) | -2.9% (-8.0 to 2.3) |
| Colorado | 604 (470 to 723) | 6.9 (5.4 to 8.2) | 66.4% (42.5 to 91.0) | 22354 (17561 to 27919) | 262.4 (206.2 to 327.4) | 16.8% (9.5 to 25.1) |
| Connecticut | 351 (275 to 423) | 4.1 (3.3 to 5.0) | 45.3% (22.7 to 68.6) | 13750 (10759 to 17387) | 193.7 (150.8 to 245.4) | 11.3% (4.6 to 19.0) |
| Delaware | 71 (58 to 84) | 3.7 (3.1 to 4.4) | 10.7% (-3.1 to 25.0) | 3405 (2651 to 4317) | 193.1 (151.1 to 243.5) | 1.1% (-3.8 to 6.3) |
| District of Columbia | 42 (32 to 50) | 3.8 (3.0 to 4.5) | 19.9% (2.2 to 39.3) | 1818 (1429 to 2331) | 193.8 (151.9 to 248.1) | -1.4% (-7.2 to 4.8) |
| Florida | 2257 (1757 to 2721) | 4.3 (3.4 to 5.2) | 47.7% (26.3 to 70.2) | 92401 (71783 to 117045) | 212.9 (166.0 to 267.1) | 12.2% (5.4 to 20.5) |
| Georgia | 750 (608 to 890) | 4.9 (4.0 to 5.8) | 17.2% (1.7 to 34.6) | 33304 (26477 to 41751) | 215.1 (170.7 to 269.6) | 0.0% (-6.6 to 6.5) |
| Hawaii | 125 (97 to 151) | 3.5 (2.8 to 4.2) | 16.6% (-0.6 to 35.9) | 5536 (4268 to 7101) | 192.3 (149.0 to 247.6) | 6.1% (0.4 to 12.0) |
| Idaho | 165 (132 to 196) | 5.7 (4.6 to 6.8) | 29.0% (10.9 to 48.2) | 6519 (5137 to 8174) | 237.7 (187.8 to 297.0) | 4.0% (-2.7 to 11.0) |
| Illinois | 898 (732 to 1075) | 3.6 (3.0 to 4.4) | 24.4% (7.1 to 44.7) | 42303 (32707 to 54191) | 192.4 (148.8 to 246.2) | 2.7% (-2.5 to 9.2) |
| Indiana | 538 (427 to 635) | 4.4 (3.5 to 5.2) | 33.0% (14.4 to 53.9) | 22821 (17969 to 28818) | 205.6 (162.3 to 260.2) | 9.0% (2.6 to 16.1) |
| Iowa | 387 (309 to 465) | 5.5 (4.4 to 6.6) | 54.3% (32.4 to 80.4) | 14109 (11095 to 17688) | 238.4 (187.9 to 298.0) | 19.1% (12.0 to 28.0) |
| Kansas | 305 (239 to 361) | 5.3 (4.2 to 6.3) | 60.0% (36.0 to 85.8) | 11376 (8974 to 14224) | 228.2 (180.0 to 284.0) | 18.4% (10.8 to 27.8) |
| Kentucky | 427 (348 to 507) | 5.4 (4.4 to 6.4) | 42.9% (22.7 to 64.1) | 17917 (14225 to 22481) | 238.8 (189.4 to 297.9) | 13.2% (5.6 to 21.6) |
| Louisiana | 323 (260 to 382) | 4.2 (3.4 to 5.1) | 21.4% (4.0 to 41.1) | 14428 (11428 to 18000) | 201.0 (159.3 to 249.1) | 4.6% (-2.8 to 13.0) |
| Maine | 160 (129 to 189) | 5.2 (4.2 to 6.1) | 62.9% (40.8 to 88.4) | 6386 (4999 to 8065) | 227.4 (177.8 to 286.5) | 16.0% (9.5 to 23.8) |
| Maryland | 459 (361 to 545) | 4.2 (3.3 to 5.0) | 27.2% (7.2 to 47.2) | 20184 (15740 to 25458) | 200.1 (155.8 to 252.0) | 6.9% (0.5 to 13.8) |
| Massachusetts | 535 (415 to 646) | 3.7 (2.9 to 4.5) | 32.4% (13.2 to 52.8) | 23944 (18397 to 30667) | 192.3 (147.3 to 246.5) | 8.8% (3.4 to 15.1) |
| Michigan | 879 (704 to 1043) | 4.4 (3.5 to 5.2) | 37.2% (17.8 to 57.4) | 35394 (28178 to 44485) | 196.9 (156.3 to 247.3) | 10.2% (3.3 to 17.5) |
| Minnesota | 747 (598 to 891) | 6.6 (5.3 to 7.9) | 56.9% (35.4 to 81.9) | 25177 (19966 to 31809) | 254.6 (202.0 to 321.0) | 15.5% (8.5 to 23.5) |
| Mississippi | 283 (228 to 334) | 5.6 (4.5 to 6.7) | 36.6% (17.2 to 59.1) | 11689 (9221 to 14642) | 245.6 (195.3 to 305.0) | 9.8% (1.8 to 19.1) |
| Missouri | 651 (517 to 773) | 5.3 (4.3 to 6.3) | 40.9% (20.4 to 62.6) | 25935 (20515 to 32796) | 235.8 (187.0 to 296.8) | 10.7% (4.0 to 19.0) |
| Montana | 142 (113 to 169) | 6.6 (5.3 to 7.9) | 43.5% (24.2 to 66.0) | 5085 (4069 to 6264) | 260.4 (209.5 to 320.5) | 13.0% (5.7 to 21.7) |
| Nebraska | 190 (154 to 225) | 5.0 (4.1 to 5.9) | 35.0% (16.1 to 55.0) | 7467 (5896 to 9376) | 227.6 (179.6 to 285.6) | 11.4% (4.9 to 18.5) |
| Nevada | 197 (161 to 231) | 4.3 (3.5 to 5.0) | 2.1% (-12.7 to 17.5) | 9470 (7488 to 11874) | 198.4 (156.7 to 248.3) | -1.1% (-7.6 to 5.4) |
| New Hampshire | 147 (116 to 176) | 5.4 (4.2 to 6.5) | 66.4% (42.0 to 93.4) | 5892 (4653 to 7443) | 229.5 (181.3 to 290.5) | 17.1% (10.6 to 24.6) |
| New Jersey | 567 (451 to 681) | 3.0 (2.4 to 3.6) | 10.0% (-6.0 to 27.5) | 27288 (21194 to 34951) | 168.0 (130.0 to 214.3) | 1.2% (-4.0 to 7.2) |
| New Mexico | 287 (230 to 341) | 6.8 (5.4 to 8.0) | 42.5% (23.6 to 63.6) | 10284 (8138 to 12993) | 268.3 (213.5 to 338.0) | 12.4% (5.7 to 20.5) |
| New York | 1246 (983 to 1507) | 3.0 (2.4 to 3.6) | 9.7% (-6.2 to 26.5) | 60353 (46903 to 76896) | 168.6 (131.8 to 214.4) | -0.7% (-5.5 to 4.7) |
| North Carolina | 995 (798 to 1173) | 5.4 (4.4 to 6.4) | 40.2% (21.0 to 62.6) | 40561 (32426 to 50705) | 232.1 (186.1 to 288.8) | 10.6% (3.8 to 18.7) |
| North Dakota | 78 (61 to 92) | 4.9 (4.0 to 5.8) | 35.5% (17.7 to 51.6) | 2917 (2287 to 3666) | 228.3 (179.4 to 286.4) | 14.3% (8.0 to 20.9) |
| Ohio | 1133 (920 to 1341) | 4.8 (3.9 to 5.7) | 44.6% (25.5 to 66.3) | 46514 (36771 to 57663) | 220.5 (173.6 to 272.8) | 15.2% (8.9 to 22.6) |
| Oklahoma | 428 (343 to 508) | 6.1 (5.0 to 7.3) | 67.5% (43.2 to 93.0) | 16199 (12796 to 20143) | 251.7 (198.4 to 313.0) | 22.2% (13.5 to 32.3) |
| Oregon | 485 (384 to 575) | 5.9 (4.7 to 6.9) | 49.9% (29.1 to 73.7) | 17888 (14079 to 22606) | 238.5 (187.9 to 301.3) | 12.2% (5.5 to 20.2) |
| Pennsylvania | 1320 (1048 to 1574) | 4.5 (3.6 to 5.3) | 43.4% (22.4 to 66.1) | 52021 (40437 to 65893) | 206.1 (160.2 to 260.8) | 13.8% (7.1 to 22.0) |
| Rhode Island | 117 (90 to 139) | 4.8 (3.8 to 5.6) | 54.0% (31.9 to 77.1) | 4398 (3465 to 5555) | 215.7 (169.5 to 273.6) | 13.5% (7.5 to 19.5) |
| South Carolina | 447 (363 to 530) | 5.1 (4.1 to 6.0) | 33.0% (14.1 to 53.2) | 18934 (14947 to 23822) | 223.0 (176.2 to 278.5) | 6.9% (-0.7 to 15.2) |
| South Dakota | 112 (91 to 131) | 6.0 (4.9 to 7.0) | 55.7% (35.7 to 77.0) | 3976 (3156 to 4950) | 256.7 (204.7 to 319.2) | 18.6% (12.1 to 26.8) |
| Tennessee | 650 (516 to 775) | 5.5 (4.3 to 6.5) | 40.2% (20.5 to 63.2) | 25780 (20206 to 32062) | 227.3 (178.1 to 282.2) | 13.0% (5.6 to 22.2) |
| Texas | 1805 (1430 to 2140) | 4.5 (3.6 to 5.3) | 29.1% (10.0 to 49.6) | 84812 (66702 to 106496) | 215.6 (169.4 to 270.1) | 6.9% (0.6 to 14.1) |
| Utah | 220 (179 to 260) | 5.7 (4.7 to 6.8) | 37.5% (19.9 to 58.3) | 8779 (6911 to 11134) | 231.9 (182.7 to 293.7) | 10.9% (4.4 to 18.1) |
| Vermont | 92 (73 to 107) | 6.4 (5.1 to 7.4) | 46.5% (29.7 to 65.1) | 3201 (2531 to 4039) | 250.5 (197.9 to 315.1) | 11.7% (6.7 to 17.4) |
| Virginia | 710 (563 to 849) | 4.8 (3.8 to 5.7) | 31.6% (11.1 to 49.9) | 29351 (23536 to 36884) | 208.8 (167.5 to 263.0) | 8.3% (1.5 to 15.1) |
| Washington | 678 (532 to 813) | 5.1 (4.0 to 6.1) | 42.3% (21.2 to 63.7) | 27431 (21354 to 34844) | 222.2 (173.8 to 281.7) | 7.3% (1.4 to 13.8) |
| West Virginia | 233 (189 to 275) | 5.9 (4.8 to 6.9) | 60.7% (38.3 to 85.4) | 8759 (6913 to 10872) | 241.2 (192.2 to 298.7) | 19.7% (11.3 to 29.8) |
| Wisconsin | 839 (669 to 1006) | 6.9 (5.5 to 8.2) | 87.3% (60.8 to 115.8) | 28116 (22092 to 35191) | 261.2 (204.9 to 327.1) | 24.6% (16.9 to 34.4) |
| Wyoming | 60 (49 to 69) | 5.6 (4.6 to 6.4) | 23.7% (10.2 to 38.2) | 2432 (1932 to 3051) | 244.2 (193.9 to 304.8) | 6.1% (0.9 to 11.8) |

Data in parentheses are 95% uncertainty intervals. DALYs= disability-adjusted life-years.

**Table S7.** Deaths and DALYs attributable to kidney dysfunction in 2019 and percentage change of age-standardised rates from 1990 to 2019, by location.

|  | Deaths |  |  | DALYs |  |  |
| --- | --- | --- | --- | --- | --- | --- |
|  | Counts | Age-standardised rates per 100,000 people | Percentage change in age-standardised rates, 1990-2019 | Counts | Age-standardised rates per 100,000 people | Percentage change in age-standardised rates, 1990-2019 |
| United States | 214741 (182315 to 248845) | 34.8 (30.0 to 39.8) | -15.0% (-20.9 to -7.0) | 3934538 (3485722 to 4406716) | 724.7 (646.8 to 808.1) | -7.9% (-13.7 to -0.9) |
| Alabama | 4236 (3490 to 5081) | 47.2 (38.9 to 56.7) | -0.8% (-15.0 to 16.3) | 82558 (70066 to 97906) | 1014.2 (860.8 to 1196.6) | 5.9% (-8.0 to 22.5) |
| Alaska | 269 (220 to 317) | 28.8 (23.5 to 34.0) | -32.0% (-41.0 to -21.1) | 5911 (5053 to 6860) | 594.4 (511.3 to 684.7) | -17.3% (-27.0 to -5.6) |
| Arizona | 4250 (3356 to 5213) | 30.3 (24.1 to 37.2) | -12.3% (-24.7 to 3.2) | 78650 (65303 to 93809) | 645.8 (539.9 to 766.2) | -3.7% (-15.7 to 10.5) |
| Arkansas | 2603 (2128 to 3158) | 45.4 (37.0 to 54.8) | 0.8% (-14.4 to 18.5) | 48871 (41032 to 57887) | 969.8 (816.5 to 1146.0) | 8.7% (-6.2 to 25.6) |
| California | 20295 (16457 to 24608) | 28.6 (23.4 to 34.5) | -20.5% (-32.5 to -5.6) | 373420 (316431 to 436330) | 603.8 (513.4 to 704.4) | -11.1% (-22.7 to 2.8) |
| Colorado | 2437 (1978 to 2944) | 27.7 (22.4 to 33.5) | -13.2% (-27.7 to 2.9) | 45573 (37919 to 53867) | 544.6 (454.9 to 642.5) | -7.7% (-20.8 to 7.1) |
| Connecticut | 2271 (1769 to 2791) | 27.6 (21.7 to 33.8) | -22.7% (-35.4 to -7.2) | 38124 (31250 to 45252) | 557.8 (461.7 to 658.9) | -17.3% (-28.8 to -3.5) |
| Delaware | 717 (590 to 868) | 36.8 (30.5 to 44.5) | -20.5% (-30.3 to -9.3) | 13183 (11216 to 15291) | 759.9 (647.1 to 876.5) | -12.2% (-21.8 to -1.6) |
| District of Columbia | 407 (326 to 493) | 39.5 (32.0 to 47.8) | -13.3% (-25.1 to 1.5) | 8310 (6989 to 9778) | 928.4 (782.4 to 1092.1) | -15.3% (-25.6 to -2.4) |
| Florida | 15511 (12259 to 19150) | 30.0 (23.8 to 36.7) | -12.8% (-26.0 to 3.9) | 273380 (226723 to 326164) | 661.9 (551.3 to 790.5) | -1.8% (-14.8 to 13.3) |
| Georgia | 6735 (5554 to 8060) | 43.5 (35.9 to 51.8) | -10.6% (-23.8 to 4.7) | 139216 (118061 to 162753) | 909.6 (772.3 to 1064.0) | -6.9% (-19.4 to 7.5) |
| Hawaii | 862 (669 to 1036) | 25.3 (20.1 to 30.2) | -15.2% (-27.0 to -0.2) | 15897 (13188 to 18498) | 589.9 (492.1 to 687.6) | -3.6% (-15.0 to 10.0) |
| Idaho | 910 (733 to 1097) | 30.9 (25.0 to 37.3) | -7.6% (-20.2 to 9.2) | 16327 (13671 to 19301) | 597.9 (502.0 to 707.5) | -3.3% (-15.3 to 12.4) |
| Illinois | 8812 (7113 to 10509) | 35.9 (29.4 to 42.9) | -20.1% (-32.0 to -4.6) | 158559 (133797 to 186228) | 741.3 (624.1 to 869.8) | -14.2% (-25.7 to 0.1) |
| Indiana | 5174 (4224 to 6221) | 42.5 (34.7 to 51.0) | -6.6% (-20.6 to 11.1) | 93967 (79596 to 110592) | 861.2 (734.2 to 1012.7) | 1.2% (-12.6 to 18.2) |
| Iowa | 2110 (1666 to 2604) | 30.2 (24.2 to 37.4) | -9.7% (-23.4 to 7.2) | 34383 (28531 to 41623) | 600.7 (497.7 to 719.0) | -1.8% (-15.2 to 13.8) |
| Kansas | 1994 (1590 to 2384) | 35.4 (28.4 to 42.1) | -2.6% (-17.8 to 14.7) | 34761 (29051 to 40853) | 715.7 (599.3 to 842.4) | 3.8% (-11.0 to 20.5) |
| Kentucky | 3719 (3046 to 4463) | 46.3 (38.0 to 55.7) | -0.5% (-14.9 to 17.9) | 70602 (59239 to 82931) | 954.1 (802.1 to 1117.6) | 5.6% (-8.6 to 22.7) |
| Louisiana | 3829 (3123 to 4581) | 49.1 (40.2 to 58.7) | -7.3% (-21.2 to 9.3) | 76499 (64593 to 89546) | 1079.1 (916.1 to 1262.3) | 0.1% (-13.5 to 16.0) |
| Maine | 1036 (835 to 1249) | 33.4 (27.1 to 40.2) | -13.0% (-25.2 to 2.6) | 17608 (14716 to 20544) | 640.7 (538.6 to 746.7) | -8.8% (-20.4 to 5.8) |
| Maryland | 3893 (3090 to 4731) | 35.8 (28.5 to 43.5) | -19.4% (-31.3 to -5.9) | 73914 (61801 to 87615) | 751.3 (629.6 to 887.4) | -12.0% (-24.0 to 1.2) |
| Massachusetts | 4442 (3492 to 5437) | 31.2 (24.8 to 38.1) | -20.0% (-32.5 to -4.7) | 74180 (61045 to 87567) | 614.6 (507.5 to 725.8) | -17.3% (-28.7 to -4.1) |
| Michigan | 7689 (6163 to 9365) | 38.3 (31.0 to 46.5) | -14.0% (-27.1 to 0.7) | 135898 (113996 to 160324) | 775.2 (651.5 to 913.6) | -8.1% (-21.0 to 6.4) |
| Minnesota | 3043 (2439 to 3646) | 27.7 (22.5 to 33.4) | -15.5% (-28.5 to 2.0) | 50661 (42364 to 59406) | 532.5 (446.6 to 622.5) | -10.5% (-22.7 to 4.0) |
| Mississippi | 2702 (2231 to 3188) | 52.4 (43.4 to 61.9) | -0.5% (-15.3 to 16.9) | 54105 (46041 to 63388) | 1152.3 (979.2 to 1347.4) | 7.7% (-7.4 to 25.3) |
| Missouri | 4796 (3840 to 5819) | 39.3 (31.4 to 47.4) | -8.0% (-21.3 to 9.6) | 86654 (72115 to 102033) | 811.7 (677.8 to 955.8) | 0.4% (-12.5 to 17.3) |
| Montana | 643 (521 to 781) | 29.3 (23.9 to 35.6) | -11.6% (-24.2 to 3.5) | 11413 (9587 to 13515) | 591.4 (497.6 to 696.9) | -4.0% (-16.3 to 10.4) |
| Nebraska | 1200 (977 to 1440) | 31.6 (26.1 to 37.8) | -14.4% (-27.3 to 1.9) | 20218 (17217 to 23534) | 630.6 (536.7 to 734.4) | -8.3% (-20.5 to 6.1) |
| Nevada | 1807 (1451 to 2187) | 38.4 (30.7 to 46.4) | -32.5% (-41.7 to -21.9) | 36014 (30106 to 42778) | 758.1 (633.9 to 895.3) | -19.5% (-30.0 to -8.5) |
| New Hampshire | 841 (661 to 1024) | 30.7 (24.3 to 37.5) | -17.0% (-29.5 to -2.1) | 14502 (11962 to 17131) | 580.2 (480.6 to 682.7) | -14.9% (-26.3 to -0.7) |
| New Jersey | 6177 (4956 to 7704) | 33.0 (26.6 to 41.1) | -23.2% (-34.7 to -9.5) | 106216 (88917 to 126824) | 671.8 (563.6 to 798.6) | -17.9% (-29.0 to -5.2) |
| New Mexico | 1362 (1107 to 1637) | 32.2 (26.3 to 38.5) | -7.0% (-19.8 to 8.5) | 25585 (21585 to 29841) | 689.1 (582.6 to 799.8) | 3.7% (-9.3 to 18.4) |
| New York | 12647 (9669 to 15795) | 30.0 (23.4 to 37.4) | -29.6% (-40.3 to -17.8) | 217210 (176875 to 259728) | 624.7 (517.5 to 742.3) | -24.2% (-34.2 to -13.2) |
| North Carolina | 7295 (5914 to 8811) | 39.6 (32.3 to 48.1) | -13.2% (-25.9 to 3.7) | 141808 (119139 to 167995) | 830.4 (698.9 to 981.7) | -7.9% (-20.8 to 6.5) |
| North Dakota | 481 (384 to 581) | 30.6 (25.1 to 36.4) | -11.8% (-22.9 to 1.3) | 7873 (6676 to 9170) | 631.3 (538.2 to 734.3) | -1.9% (-13.1 to 11.1) |
| Ohio | 9404 (7579 to 11396) | 40.2 (32.4 to 48.6) | -10.2% (-22.7 to 4.1) | 169082 (141627 to 198625) | 827.0 (696.3 to 967.4) | -2.7% (-15.1 to 11.3) |
| Oklahoma | 3115 (2460 to 3778) | 44.3 (35.2 to 53.7) | 0.1% (-14.5 to 16.6) | 58026 (48008 to 68792) | 920.1 (762.7 to 1085.2) | 7.8% (-6.7 to 23.9) |
| Oregon | 2321 (1848 to 2792) | 28.1 (22.5 to 33.8) | -19.5% (-31.5 to -5.0) | 40981 (34526 to 48268) | 558.6 (472.6 to 656.8) | -12.8% (-24.4 to 0.6) |
| Pennsylvania | 10759 (8647 to 13068) | 36.7 (29.5 to 44.3) | -18.5% (-30.0 to -3.5) | 181385 (152275 to 215908) | 741.2 (625.0 to 878.2) | -13.6% (-24.9 to 0.4) |
| Rhode Island | 727 (571 to 906) | 30.9 (24.6 to 37.9) | -18.6% (-30.3 to -4.8) | 11833 (9857 to 13925) | 606.4 (510.7 to 710.4) | -15.0% (-26.1 to -1.9) |
| South Carolina | 3816 (3118 to 4550) | 42.0 (34.4 to 50.1) | -10.7% (-24.4 to 5.4) | 76050 (63883 to 88642) | 904.9 (759.6 to 1053.6) | -5.9% (-18.9 to 9.5) |
| South Dakota | 569 (460 to 687) | 30.9 (25.5 to 36.9) | -10.0% (-21.8 to 4.2) | 9553 (8090 to 11209) | 634.9 (540.8 to 742.7) | -1.7% (-13.2 to 11.7) |
| Tennessee | 5059 (4101 to 6136) | 42.0 (34.1 to 50.9) | -7.4% (-19.9 to 9.0) | 97407 (81204 to 115728) | 877.0 (733.5 to 1034.9) | -1.2% (-13.3 to 14.7) |
| Texas | 14828 (12030 to 18163) | 36.5 (29.6 to 44.7) | -3.7% (-20.0 to 13.8) | 299173 (249461 to 354475) | 768.7 (646.1 to 909.3) | 5.6% (-9.0 to 22.1) |
| Utah | 1327 (1093 to 1599) | 34.3 (28.3 to 41.3) | -2.2% (-15.8 to 14.0) | 24069 (20453 to 28570) | 637.4 (542.7 to 756.8) | -0.2% (-12.7 to 13.9) |
| Vermont | 382 (309 to 461) | 26.9 (22.1 to 32.3) | -25.2% (-33.7 to -14.3) | 6506 (5495 to 7562) | 526.8 (448.2 to 610.9) | -19.2% (-27.4 to -9.6) |
| Virginia | 5423 (4374 to 6526) | 36.7 (29.7 to 44.1) | -16.9% (-29.8 to -2.2) | 104096 (88245 to 122238) | 758.0 (643.5 to 888.4) | -11.0% (-23.3 to 2.8) |
| Washington | 3503 (2825 to 4238) | 26.5 (21.4 to 32.1) | -21.0% (-32.6 to -5.6) | 64215 (53743 to 76507) | 531.3 (446.2 to 629.9) | -13.4% (-24.7 to 0.2) |
| West Virginia | 1929 (1573 to 2313) | 47.7 (39.0 to 57.2) | 3.3% (-11.8 to 21.6) | 34793 (29350 to 40870) | 974.0 (824.7 to 1141.4) | 8.2% (-7.0 to 25.0) |
| Wisconsin | 4070 (3294 to 4965) | 34.3 (28.0 to 41.7) | -14.7% (-28.2 to 1.3) | 69560 (58226 to 82279) | 672.4 (564.0 to 791.8) | -8.5% (-21.6 to 5.4) |
| Wyoming | 313 (259 to 368) | 28.7 (23.8 to 33.7) | -15.5% (-25.3 to -2.5) | 5761 (4951 to 6671) | 581.1 (503.1 to 669.4) | -7.8% (-17.6 to 3.9) |

Data in parentheses are 95% uncertainty intervals. DALYs= disability-adjusted life-years.

**Table S8.** Age-standardized death and DALYs rate attributable to the individual and combined effects of metabolic risks in the United States by diseases.

| Causes | Age-standardized death rates (per 100 000) | Age-standardized DALY rates (per 100 000) |
| --- | --- | --- |
| **Metabolic risks combined** | | |
| Neoplasms | 19.21 (10.63 to 29.94) | 422.70(244.37 to 644.74) |
| Cardiovascular diseases | 112.55 (99.65 to 123.67) | 2294.31 (2112.93 to 2476.98) |
| Chronic respiratory diseases | 0.31 (0.21 to 0.43) | 113.272 (70.88 to 171.41) |
| Digestive diseases | 0.53 (0.33 to 0.72) | 34.64 (21.71 to 51.20) |
| Neurological disorders | 6.28 (1.13 to 18.60) | 98.28 (31.57 to 230.53) |
| Diabetes mellitus | 13.69 (12.83 to 14.17) | 875.82 (702.80 to 1079.61) |
| Chronic kidney disease | 17.77 (16.11 to 18.91) | 438.19 (402.09 to 477.33) |
| Injuries | 4.47 (3.76 to 4.94) | 210.335 (166.33 to 263.83) |
| Respiratory infections and tuberculosis | 0.03 (0.02 to 0.04) | 0.78 (0.50 to 1.08) |
| Musculoskeletal disorders | ― | 245.86 (143.39 to 374.04) |
| Sense organ diseases | ― | 4.49 (2.09 to 8.00) |
| **High body mass index** | | |
| Neoplasms | 9.74 (6.09 to 13.59) | 232.05 (147.59 to 318.60) |
| Cardiovascular diseases | 39.00 (25.52 to 52.37) | 1047.59 (731.14 to 1330.83) |
| Chronic respiratory diseases | 0.31 (0.21 to 0.43) | 113.27 (70.88 to 171.41) |
| Digestive diseases | 0.53 (0.33 to 0.72) | 34.64 (21.71 to 51.20) |
| Neurological disorders | 4.08 (0.68 to 11.91) | 64.54 (19.00 to 152.85) |
| Diabetes mellitus | 7.53 (5.56 to 9.38) | 566.17 (406.03 to 742.58) |
| Chronic kidney disease | 7.34 (4.29 to 10.44) | 196.65 (132.45 to 260.86) |
| Musculoskeletal disorders | ― | 241.26 (138.35 to 370.98) |
| Sense organ diseases | ― | 2.00 (0.96 to 3.45) |
| **High fast plasma glucose** | | |
| Neoplasms | 9.97 (2.86 to 19.61) | 201.36 (57.46 to 395.30) |
| Cardiovascular diseases | 40.39 (26.59 to 59.67) | 739.33 (542.46 to 994.11) |
| Neurological disorders | 2.73 (0.32 to 10.03) | 42.32 (7.92 to 131.47) |
| Diabetes mellitus | 13.69 (12.83 to 14.17) | 875.82 (702.80 to 1079.61) |
| Chronic kidney disease | 5.91 (4.60 to 7.22) | 140.99 (115.10 to 166.77) |
| Respiratory infections and tuberculosis | 0.03 (0.02 to 0.04) | 0.78 (0.50 to 1.08) |
| Sense organ diseases | ― | 2.73 (0.71 to 6.12) |
| High LDL cholesterol |  |  |
| Cardiovascular diseases | 38.02 (27.73 to 50.01) | 804.95 (656.28 to 968.29) |
| **High Systolic blood pressure** | | |
| Cardiovascular diseases | 70.31 (58.00 to 82.57) | 1448.71 (1254.69 to 1640.65) |
| Chronic kidney disease | 11.33 (9.80 to 12.71) | 235.42 (205.82 to 264.92) |
| **Kidney dysfunction** | | |
| Cardiovascular diseases | 17.05 (12.80 to 21.42) | 277.8241 (219.66 to 337.93) |
| Chronic kidney disease | 17.77 (16.12 to 18.91) | 438.1982 (402.09 to 477.34) |
| Musculoskeletal disorders | ― | 8.66387 (5.73 to 12.27) |
| **Low bone mineral density** | | |
| Injuries | 4.47 (3.76 to 4.95) | 210.34 (166.33 to 263.83) |

**Table S9.** Age-standardized proportion of deaths attributable to the individual and combined effects of metabolic risks in the United States, 2019.

|  | PAF of risk factor (95% UI) | | | | | | |
| --- | --- | --- | --- | --- | --- | --- | --- |
|  | High body mass index | High fasting plasma glucose | High LDL cholesterol | High systolic blood pressure | Kidney dysfunction | Low bone mineral density | Metabolic risks (six risk factors combined) |
| **All causes** | 13.0% (8.7 to 17.1) | 13.8% (10.3 to 18.0) | 7.2% (5.2 to 9.4) | 15.5% (13.0 to 17.9) | 6.6% (5.7 to 7.6) | 0.8% (0.7 to 0.9) | 33.1% (29.6 to 36.6) |
| **Cardiovascular diseases** | 24.9% (16.4 to 33.0) | 25.7% (17.2 to 37.1) | 24.2% (18.0 to 31.3) | 44.8% (37.4 to 51.9) | 10.9% (8.2 to 13.6) | ― | 71.7% (65.9 to 76.8) |
| Rheumatic heart disease | ― | ― | ― | 21.8% (13.0 to 40.3) | ― | ― | 21.8% (13.0 to 40.3) |
| Ischemic heart disease | 27.0% (16.9 to 37.0) | 33.8% (19.5 to 52.3) | 38.7% (28.4 to 50.1) | 45.5% (34.8 to 56.5) | 16.0% (11.4 to 20.6) | ― | 82.6% (75.1 to 89.1) |
| Stroke | 24.4% (16.3 to 32.4) | 28.7% (17.9 to 46.8) | 9.1% (2.3 to 21.5) | 43.5% (34.7 to 53.0) | 6.8% (4.0 to 9.5) | ― | 66.1% (56.5 to 76.3) |
| Hypertensive heart disease | 60.0% (38.9 to 80.0) | ― | ― | 100.0% (100.0 to 100.0) | ― | ― | 100.0% (100.0 to 100.0) |
| Cardiomyopathy and myocarditis | ― | ― | ― | 21.9% (16.5 to 28.1) | ― | ― | 21.9% (16.5 to 28.1) |
| Atrial fibrillation and flutter | 30.0% (17.6 to 44.3) | ― | ― | 30.2% (23.0 to 37.6) | ― | ― | 45.3% (34.9 to 56.1) |
| Aortic aneurysm | ― | ― | ― | 29.0% (22.0 to 35.9) | ― | ― | 29.0% (22.0 to 35.9) |
| Peripheral artery disease | ― | 33.7% (28.7 to 38.3) | ― | 21.8% (15.2 to 29.8) | 15.3% (9.7 to 20.3) | ― | 55.8% (49.6 to 61.8) |
| Endocarditis | ― | ― | ― | 28.9% (21.7 to 37.7) | ― | ― | 28.9% (21.7 to 37.7) |
| Non-rheumatic valvular heart disease | ― | ― | ― | 23.3% (15.7 to 34.3) | ― | ― | 23.3% (15.7 to 34.3) |
| Other cardiovascular and circulatory diseases | ― | ― | ― | 22.1% (18.1 to 28.2) | ― | ― | 22.1% (18.1 to 28.2) |
| **Diabetes and kidney diseases** | 47.2% (33.1 to 60.5) | 62.2% (58.3 to 66.3) | ― | 36.0% (32.3 to 39.4) | 56.4% (55.4 to 57.4) | ― | 99.9% (99.8 to 99.9) |
| Diabetes mellitus | 55.0% (40.8 to 67.9) | 100.0% (100.0 to 100.0) | ― | ― | ― | ― | 100.0% (100.0 to 100.0) |
| Chronic kidney disease | 41.3% (24.2 to 58.6) | 33.3% (26.4 to 40.5) | ― | 63.8% (57.4 to 69.7) | 100.0% (100.0 to 100.0) | ― | 100.0% (100.0 to 100.0) |
| **Neoplasms** | 7.1% (4.4 to 9.8) | 7.3% (2.1 to 14.2) | ― | ― | ― | ― | 14.0% (7.8 to 21.7) |
| Esophageal cancer | 34.6% (11.7 to 56.9) | ― | ― | ― | ― | ― | 34.6% (11.7 to 56.9) |
| Liver cancer | 23.3% (10.6 to 38.1) | 2.3% (0.6 to 4.7) | ― | ― | ― | ― | 25.3% (12.5 to 40.2) |
| Tracheal, bronchus, and lung cancer | ― | 13.2% (3.3 to 27.1) | ― | ― | ― | ― | 13.2% (3.3 to 27.1) |
| Breast cancer | 6.9% (1.7 to 13.1) | 10.1% (2.0 to 21.4) | ― | ― | ― | ― | 16.1% (6.3 to 28.5) |
| Uterine cancer | 54.5% (39.3 to 68.0) | ― | ― | ― | ― | ― | 54.5% (39.3 to 68.0) |
| Colon and rectum cancer | 13.1% (7.6 to 18.6) | 12.7% (3.2 to 26.4) | ― | ― | ― | ― | 24.0% (13.4 to 37.1) |
| Gallbladder and biliary tract cancer | 27.0% (16.0 to 39.7) | ― | ― | ― | ― | ― | 27.0% (16.0 to 39.7) |
| Pancreatic cancer | 9.3% (3.6 to 16.6) | 12.9% (3.1 to 26.7) | ― | ― | ― | ― | 21.0% (9.8 to 35.3) |
| Ovarian cancer | 4.7% (-0.1 to 10.4) | 11.0% (2.3 to 24.5) | ― | ― | ― | ― | 15.2% (4.9 to 29.0) |
| Kidney cancer | 27.0% (16.6 to 37.6) | ― | ― | ― | ― | ― | 27.0% (16.6 to 37.6) |
| Bladder cancer | ― | 14.1% (3.0 to 29.1) | ― | ― | ― | ― | 14.1% (3.0 to 29.1) |
| Thyroid cancer | 18.4% (9.5 to 28.5) | ― | ― | ― | ― | ― | 18.4% (9.5 to 28.5) |
| Non-Hodgkin lymphoma | 8.8% (3.9 to 14.9) | ― | ― | ― | ― | ― | 8.8% (3.9 to 14.9) |
| Multiple myeloma | 10.1% (4.7 to 16.8) | ― | ― | ― | ― | ― | 10.1% (4.7 to 16.8) |
| Leukemia | 10.9% (5.7 to 17.0) | ― | ― | ― | ― | ― | 10.9% (5.7 to 17.0) |
| **Injuries** | ― | ― | ― | ― | ― | 10.3% (8.8 to 11.3) | 10.3% (8.8 to 11.3) |
| Road injuries | ― | ― | ― | ― | ― | 7.9% (6.7 to 8.6) | 7.9% (6.7 to 8.6) |
| Other transport injuries | ― | ― | ― | ― | ― | 13.5% (11.6 to 14.7) | 13.5% (11.6 to 14.7) |
| Falls | ― | ― | ― | ― | ― | 46.8% (40.7 to 50.3) | 46.8% (40.7 to 50.3) |
| Exposure to mechanical forces | ― | ― | ― | ― | ― | 8.6% (7.4 to 9.3) | 8.6% (7.4 to 9.3) |
| Animal contact | ― | ― | ― | ― | ― | 8.3% (7.2 to 9.0) | 8.3% (7.2 to 9.0) |
| Interpersonal violence | ― | ― | ― | ― | ― | 0.4% (0.4 to 0.5) | 0.4% (0.4 to 0.5) |
| **Other causes** |  |  |  |  |  |  |  |
| Tuberculosis | ― | 16.5% (9.8 to 23.8) | ― | ― | ― |  | 16.5% (9.8 to 23.8) |
| Asthma | 36.0% (23.5 to 48.7) | ― | ― | ― | ― | ― | 36.0% (23.5 to 48.7) |
| Gallbladder and biliary diseases | 47.3% (31.0 to 62.5) | ― | ― | ― | ― | ― | 47.3% (31.0 to 62.5) |
| Alzheimer's disease and other dementias | 19.4% (7.8 to 34.2) | 13.0% (3.1 to 27.1) | ― | ― | ― | ― | 29.9% (14.2 to 47.9) |

PAF, population attributable fraction; UI, uncertainty interval; LDL, low-density lipoprotein

**Table S10.** Age-standardized proportion of DALYs attributable to the individual and combined effects of metabolic risks in the United States, 2019.

|  | PAF of risk factor (95% UI) | | | | | | |
| --- | --- | --- | --- | --- | --- | --- | --- |
|  | High body mass index | High fasting plasma glucose | High LDL cholesterol | High systolic blood pressure | Kidney dysfunction | Low bone mineral density | Metabolic risks (six risk factors combined) |
| **All causes** | 9.6% (6.9 to 12.0) | 7.7% (6.5 to 9.2) | 3.1% (2.4 to 3.8) | 6.5% (5.5 to 7.5) | 2.8% (2.4 to 3.2) | 0.8% (0.7 to 0.9) | 18.2% (16.3 to 20.3) |
| **Cardiovascular diseases** | 32.5% (22.8 to 41.1) | 23.0% (17.2 to 30.6) | 25.0% (20.4 to 29.8) | 45.0% (39.7 to 50.2) | 8.6% (6.8 to 10.4) | ― | 71.2% (66.6 to 75.6) |
| Rheumatic heart disease | ― | ― | ― | 21.3% (14.2 to 34.3) | ― | ― | 21.3% (14.2 to 34.3) |
| Ischemic heart disease | 35.1% (23.4 to 46.1) | 32.2% (21.3 to 46.0) | 44.8% (36.8 to 53.5) | 46.1% (37.7 to 54.3) | 13.5% (10.0 to 17.1) | ― | 84.7% (79.1 to 90.0) |
| Stroke | 39.0% (28.7 to 47.9) | 28.1% (19.4 to 41.1) | 10.5% (5.6 to 18.2) | 44.8% (37.6 to 51.9) | 7.0% (5.2 to 8.6) | ― | 69.5% (62.3 to 76.3) |
| Hypertensive heart disease | 68.2% (51.3 to 82.5) | ― | ― | 100.0% (100.0 to 100.0) | ― | ― | 100.0% (100.0 to 100.0) |
| Cardiomyopathy and myocarditis | ― | ― | ― | 19.1% (14.8 to 23.4) | ― | ― | 19.1% (14.8 to 23.4) |
| Atrial fibrillation and flutter | 32.3% (19.2 to 46.3) | ― | ― | 33.4% (27.8 to 38.9) | ― | ― | 49.0% (39.4 to 59.3) |
| Aortic aneurysm | ― | ― | ― | 30.3% (23.9 to 36.4) | ― | ― | 30.3% (23.9 to 36.4) |
| Peripheral artery disease | ― | 36.1% (32.2 to 39.9) | ― | 22.4% (16.9 to 28.4) | 19.0% (14.9 to 23.2) | ― | 59.7% (54.9 to 64.1) |
| Endocarditis | ― | ― | ― | 29.4% (23.2 to 36.2) | ― | ― | 29.4% (23.2 to 36.2) |
| Non-rheumatic valvular heart disease | ― | ― | ― | 20.1% (14.5 to 27.0) | ― | ― | 20.1% (14.5 to 27.0) |
| Other cardiovascular and circulatory diseases | ― | ― | ― | 23.3% (19.3 to 27.9) | ― | ― | 23.3% (19.3 to 27.9) |
| **Diabetes and kidney diseases** | 58.0% (44.6 to 68.5) | 77.2% (73.9 to 80.5) | ― | 18.0% (15.1 to 21.0) | 33.5% (29.3 to 37.6) | ― | 99.9% (99.9 to 99.9) |
| Diabetes mellitus | 64.6% (51.6 to 74.7) | 100.0% (100.0 to 100.0) | ― | ― | ― | ― | 100.0% (100.0 to 100.0) |
| Chronic kidney disease | 44.9% (30.7 to 58.2) | 32.2% (26.5 to 37.5) | ― | 53.7% (48.1 to 59.2) | 100.0% (100.0 to 100.0) | ― | 100.0% (100.0 to 100.0) |
| **Neoplasms** | 7.2% (4.6 to 9.8) | 6.2% (1.8 to 12.3) | ― | ― | ― | ― | 13.1% (7.6 to 19.8) |
| Esophageal cancer | 36.1% (12.3 to 59.2) | ― | ― | ― | ― | ― | 36.1% (12.3 to 59.2) |
| Liver cancer | 24.2% (11.1 to 39.7) | 1.9% (0.5 to 4.0) | ― | ― | ― | ― | 25.9% (12.5 to 41.1) |
| Tracheal, bronchus, and lung cancer | ― | 12.3% (3.1 to 25.4) | ― | ― | ― | ― | 12.3% (3.1 to 25.4) |
| Breast cancer | 4.1% (-0.7 to 9.4) | 8.7% (1.7 to 18.5) | ― | ― | ― | ― | 12.0% (3.2 to 22.9) |
| Uterine cancer | 56.1% (40.8 to 69.1) | ― | ― | ― | ― | ― | 56.1% (40.8 to 69.1) |
| Colon and rectum cancer | 14.2% (8.5 to 19.9) | 11.2% (2.7 to 23.5) | ― | ― | ― | ― | 23.8% (13.8 to 35.7) |
| Gallbladder and biliary tract cancer | 28.0% (16.9 to 40.7) | ― | ― | ― | ― | ― | 28.0% (16.9 to 40.7) |
| Pancreatic cancer | 9.7% (3.6 to 17.3) | 11.9% (2.9 to 24.7) | ― | ― | ― | ― | 20.5% (9.7 to 34.1) |
| Ovarian cancer | 4.9% (-0.1 to 10.8) | 9.6% (2.0 to 21.6) | ― | ― | ― | ― | 14.0% (4.4 to 26.6) |
| Kidney cancer | 27.5% (17.2 to 37.9) | ― | ― | ― | ― | ― | 27.5% (17.2 to 37.9) |
| Bladder cancer | ― | 13.3% (2.9 to 27.5) | ― | ― | ― | ― | 13.3% (2.9 to 27.5) |
| Thyroid cancer | 19.4% (10.1 to 29.9) | ― | ― | ― | ― | ― | 19.4% (10.1 to 29.9) |
| Non-Hodgkin lymphoma | 9.2% (4.2 to 15.3) | ― | ― | ― | ― | ― | 9.2% (4.2 to 15.3) |
| Multiple myeloma | 10.7% (5.1 to 17.7) | ― | ― | ― | ― | ― | 10.7% (5.1 to 17.7) |
| Leukemia | 10.2% (5.5 to 15.6) | ― | ― | ― | ― | ― | 10.2% (5.5 to 15.6) |
| **Musculoskeletal disorders** | 7.2% (4.7 to 9.8) | ― | ― | ― | 0.3% (0.2 to 0.3) | ― | 7.4% (4.9 to 9.9) |
| Osteoarthritis | 15.7% (8.9 to 22.9) | ― | ― | ― | ― | ― | 15.7% (8.9 to 22.9) |
| Low back pain | 11.1% (7.1 to 15.0) | ― | ― | ― | ― | ― | 11.1% (7.1 to 15.0) |
| Gout | 50.6% (31.2 to 70.2) | ― | ― | ― | 16.4% (14.4 to 18.7) | ― | 59.3% (42.5 to 76.0) |
| **Injuries** | ― | ― | ― | ― | ― | 7.6% (6.5 to 8.8) | 7.6% (6.5 to 8.8) |
| Road injuries | ― | ― | ― | ― | ― | 6.1% (5.0 to 6.7) | 6.1% (5.0 to 6.7) |
| Other transport injuries | ― | ― | ― | ― | ― | 16.3% (13.9 to 18.6) | 16.3% (13.9 to 18.6) |
| Falls | ― | ― | ― | ― | ― | 30.4% (26.3 to 32.9) | 30.4% (26.3 to 32.9) |
| Exposure to mechanical forces | ― | ― | ― | ― | ― | 9.6% (8.2 to 10.6) | 9.6% (8.2 to 10.6) |
| Animal contact | ― | ― | ― | ― | ― | 8.4% (6.7 to 10.2) | 8.4% (6.7 to 10.2) |
| Interpersonal violence | ― | ― | ― | ― | ― | 0.6% (0.5 to 0.7) | 0.6% (0.5 to 0.7) |
| **Other causes** |  |  |  |  |  |  |  |
| Tuberculosis | ― | 14.2% (9.1 to 19.6) | ― | ― | ― | ― | 14.2% (9.1 to 19.6) |
| Asthma | 26.1% (18.0 to 34.5) | ― | ― | ― | ― | ― | 26.1% (18.0 to 34.5) |
| Gallbladder and biliary diseases | 51.3% (36.4 to 64.9) | ― | ― | ― | ― | ― | 51.3% (36.4 to 64.9) |
| Blindness and vision loss | 2.4% (1.2 to 3.8) | 3.3% (0.9 to 6.9) | ― | ― | ― | ― | 5.4% (2.7 to 9.0) |
| Alzheimer's disease and other dementias | 20.2% (8.3 to 35.6) | 13.3% (3.2 to 27.7) | ― | ― | ― | ― | 30.8% (15.5 to 48.8) |

DALYs, disability-adjusted life years; PAF, population attributable fraction; UI, uncertainty interval; LDL, low-density lipoprotein.
